# Identifying Research Priorities in Digital Education for Health Care: Umbrella Review and Modified Delphi Method Study

**DOI:** 10.2196/66157

**Published:** 2025-02-19

**Authors:** Alison Potter, Chris Munsch, Elaine Watson, Emily Hopkins, Sofia Kitromili, Iain Cameron O'Neill, Judy Larbie, Essi Niittymaki, Catriona Ramsay, Joshua Burke, Neil Ralph

**Affiliations:** 1 Technology Enhanced Learning NHS England Southampton United Kingdom; 2 Technology Enhanced Learning NHS England Leeds United Kingdom; 3 Technology Enhanced Learning NHS England Oxford United Kingdom; 4 Knowledge Management Service NHS England Manchester United Kingdom; 5 Technology Enhanced Learning NHS England London United Kingdom; 6 Technology Enhanced Learning NHS England Newcastle upon Tyne United Kingdom; 7 Manchester Foundation Trust Manchester United Kingdom

**Keywords:** digital education, health professions education, research priorities, umbrella review, Delphi, artificial intelligence, AI

## Abstract

**Background:**

In recent years, the use of digital technology in the education of health care professionals has surged, partly driven by the COVID-19 pandemic. However, there is still a need for focused research to establish evidence of its effectiveness.

**Objective:**

This study aimed to define the gaps in the evidence for the efficacy of digital education and to identify priority areas where future research has the potential to contribute to our understanding and use of digital education.

**Methods:**

We used a 2-stage approach to identify research priorities. First, an umbrella review of the recent literature (published between 2020 and 2023) was performed to identify and build on existing work. Second, expert consensus on the priority research questions was obtained using a modified Delphi method.

**Results:**

A total of 8857 potentially relevant papers were identified. Using the PRISMA (Preferred Reporting Items for Systematic Reviews and Meta-Analyses) methodology, we included 217 papers for full review. All papers were either systematic reviews or meta-analyses. A total of 151 research recommendations were extracted from the 217 papers. These were analyzed, recategorized, and consolidated to create a final list of 63 questions. From these, a modified Delphi process with 42 experts was used to produce the top-five rated research priorities: (1) How do we measure the learning transfer from digital education into the clinical setting? (2) How can we optimize the use of artificial intelligence, machine learning, and deep learning to facilitate education and training? (3) What are the methodological requirements for high-quality rigorous studies assessing the outcomes of digital health education? (4) How does the design of digital education interventions (eg, format and modality) in health professionals’ education and training curriculum affect learning outcomes? and (5) How should learning outcomes in the field of health professions’ digital education be defined and standardized?

**Conclusions:**

This review provides a prioritized list of research gaps in digital education in health care, which will be of use to researchers, educators, education providers, and funding agencies. Additional proposals are discussed regarding the next steps needed to advance this agenda, aiming to promote meaningful and practical research on the use of digital technologies and drive excellence in health care education.

## Introduction

In just over two decades, the use of digital technology for delivering education and learning has increased globally [1]. The growth of digital education in health care has seen a similar trajectory internationally [2].

The adoption of digital technologies in health care education forms part of a global strategy to invest in the health workforce [3] and support the United Nations 2023 Agenda for Sustainable Development [4]. This agenda is echoed within the United Kingdom’s National Health Service (NHS) Long Term Workforce Plan [5]. In its plan to address projected workforce shortages, the NHS has undertaken to expand health care education and training over the next 15 years. Informed by the Topol Review [6], the Long Term Workforce Plan acknowledges the need to embrace a wide range of learning methods to achieve the scale of education delivery that will be required [5]. These methods include the use of web-based learning, blended learning, hybrid learning, and simulation and immersive technologies. In addition, it is clear that easily accessible and usable artificial intelligence (AI) will have an increasing role in education [5].

The COVID-19 pandemic saw a surge in the use of digital technologies for health care education. Consequently, several bibliometric analyses illustrate how research activity across a variety of digital technologies significantly increased internationally [2,7-13]. Since 2020, research activity has shifted from rapid development during the early stages of the COVID-19 pandemic to a focus on quality improvement and opportunities for sustained use in the postpandemic period [8]. Zhang et al [8] state that the data project a trend of continued growth in digital education in health care globally, including increased application of advanced technologies. In the United Kingdom, the Council of Deans of Health highlighted in their report “Post-Pandemic Progress: Lessons Learnt in Healthcare Education” [14] that attitudes of educators and students toward the use of digital modalities have shifted. Universities UK [15] echoes this sentiment, recommending increased investment and further expansion of digital technologies in health professions education.

To successfully leverage the full potential of existing technologies for education delivery and understand the opportunities presented by advanced technologies, there is a requirement to ensure that learning needs are being effectively met [16] and that there is proven return on the investment needed to deploy these technologies. The Immersive Healthcare Collaboration [17] emphasized the importance of rigorous evaluation when adopting new technologies and highlighted the need for continued investment in digital education research. However, currently it is not clear where these research efforts and resources should be focused.

Recent studies on research priorities in health care digital education have mostly focused on surgery [18-25]. However, a recent literature review by Tudor Car et al [26] explored evidence across all health care disciplines and across multiple digital education modalities. The authors sought to broaden the scope of their inquiry beyond evidence of effectiveness and to identify where gaps in the evidence lay around other factors such as context, infrastructure, education, and learners [26]. In doing so, they constructed an evidence map, a contextual framework, and a potential research agenda. They offered a list of research questions that, at that time, were still to be fully addressed by the scientific literature [26]. While this work is both comprehensive and informative, it was conducted before or early in the COVID-19 pandemic era; all the reviewed papers were published between January 2014 and July 2020. With the massive increase in the use of digital education in response to the COVID-19 pandemic, the authors of this study felt that this work warranted revisiting and updating to uncover additional evidence gaps and new areas of inquiry that may have emerged since 2020.

Therefore, this study aimed to build on work by Tudor Car et al [26] and identify, within the conceptual framework, any new research priorities in health care digital education that have emerged since 2020. Identifying these research priorities would help shape future global research in digital education and guide the determination of priorities for activity and resource allocation within NHS England.

## Methods

### Overview

To identify research priorities in health care digital education, a 2-stage approach was used. First, an umbrella review was conducted to build on previous work, and second, experts were invited to reach consensus on the relevant research questions using a modified Delphi method. This 2-stage approach was used to ensure a balance between the published literature and test its applicability through expert opinion. Details of each stage are described in the following sections.

### Umbrella Review

As a first step to identifying any additional research questions since the study by Tudor Car et al [26], an umbrella review (a review of systematic reviews) of the literature was conducted based on the method used by Tudor Car et al [26]. The systematic review and research syntheses checklist has been used to self-appraise the process on completion (Multimedia Appendix 1) [27].

#### Inclusion and Exclusion Criteria

Studies eligible for inclusion were peer-reviewed systematic reviews or meta-analyses with a clear focus on digital education for health professions education, which were published between 2020 and 2023 and written in English. The list of digital education technologies as described by Tudor Car et al [26] was used to guide eligibility screening. For convenience, definitions have been shared in Multimedia Appendix 2 (Textbox 1).

**Eligibility criteria.**

**Included (studies involving one or more of the following digital education technologies)**

- Online digital education
- Massive open web-based course
- Mobile education
- Serious gaming and gamification
- Virtual reality
- Virtual patients
- High-fidelity manikins
- Blended education
- Augmented reality
- Extended reality

**Excluded**

- 3D printing (after considerable discussion, it was decided that 3D printing should not be considered a digital educational intervention because this intervention leads to an analogue product and is used as such)
- Primary research
- Conference abstracts
- Non-English language papers

To further assess eligibility for inclusion, all the following questions had to be answered in the affirmative:

1. Are the participants in the intervention preservice (ie, students) or in-service professionals (ie, after degree completion, including postgraduate trainees and those in independent practice)?
2. Are the participants from disciplines such as medicine, dentistry, nursing and midwifery, medical diagnostic and treatment technology, physiotherapy and rehabilitation, and pharmacy? (practitioners of traditional, alternative, and complementary medicine were excluded)
3. Is the educational intervention using digital technology?

#### Information Sources

A knowledge specialist (EW) searched the following major bibliographic databases for review papers meeting the inclusion criteria and published between July 2020 and April 2023: MEDLINE (Ovid), Embase (Ovid), CINAHL (EBSCO), and the Cochrane Library. In addition, the knowledge specialist (EW) searched ERIC, Google Scholar, and Research Gate using the following search terms: *systematic review, digital education, eLearning, health professionals,* and *health professions education.* All searches were conducted on April 21, 2023.

#### Search Strategy

The MEDLINE (Ovid) search strategy was identical to that used by Tudor Car et al [26] (Multimedia Appendix 3). Minor modifications were needed to convert the strategy for CINAHL (EBSCO) and the Cochrane Library.

#### Study Selection

The PRISMA (Preferred Reporting Items for Systematic Reviews and Meta-Analyses) methodology was used to identify studies for inclusion. The search results were captured in RefWorks [28], and duplicates were removed. Pairs of independent reviewers screened the title and abstract of each paper against the eligibility criteria and predefined question set for inclusion. The full-text papers meeting the inclusion criteria were then retrieved and reviewed by pairs of independent reviewers. At both stages of screening, any discrepancy between reviewer pairs was resolved by a third independent reviewer.

#### Data Extraction

A data capture tool was developed in Jisc Online Surveys [29] and piloted by 8 reviewers (AP, CM, EH, SK, ION, EN and 2 volunteers from the NHS England technology enhanced learning team) using a sample paper, with refinements made to the tool before data extraction from all papers included in the review.

Data extracted were study characteristics and future research recommendations as set out by the authors. Future research recommendations were mapped to the conceptual domain framework devised by Tudor Car et al [26] and any previously identified research questions, where possible. If there was insufficient alignment with existing research questions, a new one was created. All papers with future research recommendations were reviewed a second time, with any discrepancies resolved by a third independent reviewer.

#### Analysis

AP and EW consolidated the extracted research questions by mapping them to existing questions, removing duplicates, and rephrasing those with similar meanings. New questions were then grouped and categorized by CM into overarching research questions. CM and AP discussed and reached a consensus on the final set of questions.

### Delphi Consensus

#### Overview

A 4-round modified Delphi was conducted through January to March 2024 to arrive at 5 final research questions. The method consisted of a desktop exercise to formulate the Delphi survey from the results of the umbrella review (round 1), assessment (round 2), prioritization (round 3), and ranking (round 4). Each survey was created by AP and tested by members of the research team.

#### Round 1: Umbrella Review and Preparation of the Survey

The results of the umbrella review provided the material to compile a web-based survey of research questions for consideration by experts in later rounds. Research questions, previously identified in the umbrella review, meeting the inclusion criteria formed the basis of a web-based survey in round 2.

Inclusion criteria were as follows:

- The median number of papers identified and mapped to an existing research question in the study by Tudor Car et al [26] was 7.5, and this was used as an inclusion threshold.
- Following consolidation, all newly identified questions were included in the survey.

#### Round 2: Assessment Against Criteria

A total of 42 participants were recruited, including digital education professionals and simulation faculty, clinical educators, health and care workforce development and transformation leads, academics, and health care students, using the set criteria (Textbox 2). Recruitment was advertised via the Association for Learning Technologies’ weekly news digest and a nationwide digital education mailing list for health professionals maintained by NHS England (Multimedia Appendix 4).

A web-based survey, created in JISC Online Surveys and comprising all research questions from round 1, was distributed to the expert panel.

Using an adapted set of criteria from the Delphi process used by Schneider et al [30], participants were invited to anonymously score each research question against 5 criteria (Table 1).

Research questions that met the consensus threshold formed the basis for round 3.

The consensus thresholds for Round 2 were as follows:

- Inclusion: >75% of respondents provide a positive result (3) on a 3-point Likert scale for all criteria.
- Exclusion: >75% of respondents provide a negative result (2) on a 3-point Likert scale for all criteria.
- Nonconsensus: when the proposed priority research question has met neither the inclusion nor the exclusion consensus thresholds.

Questions that met the inclusion criteria and those with nonconsensus were carried forward into round 3.

**Participant recruitment criteria per expert group.**

**Stakeholder group and inclusion criteria for expertise**

- Technology enhanced learning (TEL) professional and simulation faculty: substantial experience working in TEL or simulation in the National Health Service or academia
- Educator: anyone in clinical practice who trains health care professionals (such as consultant or nurse educator)
- Workforce development and transformation lead: experience or knowledge of TEL in health and care education within their geography or domain
- Academic: working in academia, with at least 1 first or senior author paper in the field of TEL
- Learner: health care student, either final-year undergraduate or postgraduate student or trainee

**Table 1:** Scoring options for each research question in round 2.

Criteria	Scoring options		
Scientific merit	1=unable to respond	2=disagree	3=agree
Significance for workforce development	1=unable to respond	2=disagree	3=agree
Innovation	1=unable to respond	2=disagree	3=agree
Relevance to the LTWP^a^	1=unable to respond	2=disagree	3=agree
Feasibility for further study	1=unable to respond	2=disagree	3=agree

^a^LTWP: Long Term Workforce Plan.

#### Round 3: Prioritization

Participants’ voting in this round was informed by knowledge of the consensus outcomes for each research question from round 2. A web-based survey was shared with participants, consisting of 2 sections: one with research questions that met the inclusion criteria for round 2 (including the percentage consensus agreement for each question) and another with questions from round 2 that did not reach consensus.

Participants were invited to score each research question again, but this time based on priority, with 1 score assigned to each question. The scoring options were as follows: 1=not to be studied, 2=low priority, 3=medium priority, and 4=high priority.

The consensus thresholds for Round 3 were as follows:

- Inclusion: ≥80% of respondents scored the research question as a 3 or 4
- Exclusion: 100% of respondents scored the research question as a 1 (not to be studied)
- Nonconsensus: when the proposed priority research question met neither the inclusion nor the exclusion consensus thresholds

Those meeting the inclusion criteria were taken forward for ranking in round 4.

#### Round 4: Ranking

A web-based meeting was conducted with participants, facilitated by CM and AP. Participants were sent results of the previous round in advance (Multimedia Appendix 5). The meeting offered the opportunity to discuss results from round 3 in small groups. Finally, participants were asked to select and rank their top 5 research questions independently via a web-based survey constructed in Slido [31], a web-based audience interaction tool that offers in-meeting and between-meeting polling and surveying. Participants unable to attend the meeting were invited to complete this exercise within 1 week of the meeting date. After this time, for each ranked question, points were assigned in a descending order, where higher ranks received more points, for example, a question ranked first place received 5 points, and each subsequent rank received 1 point less, down to 1 point for a question ranked fifth. The 5 research questions with the highest mean formed the final set of research priorities.

Finally, a narrative synthesis approach was chosen to summarize the diverse range of selected studies in a structured manner.

### Ethical Considerations

The NHS Research Ethics screening tool was completed, and approval was not required for this study. However, key ethical requirements were considered when designing and conducting the Delphi. The NHS England Data Protection Impact Assessment was completed. Delphi participant recruitment was voluntary, with opt out being possible. Compensation was not provided. A participant information sheet outlining the purpose of the study, key contacts, their potential involvement with anticipated time commitment, and how data would be handled and stored and by whom was shared. Personal data were stored in a spreadsheet hosted on an organizational SharePoint and were only accessible to relevant members of the research team. Participants were not asked for any personal details in the Delphi surveys; instead, they were assigned a code number. This code number allowed the research team to keep track of response rates and to administrate any follow up.

## Results

### Umbrella Review

#### Study Selection

**Figure 1:**
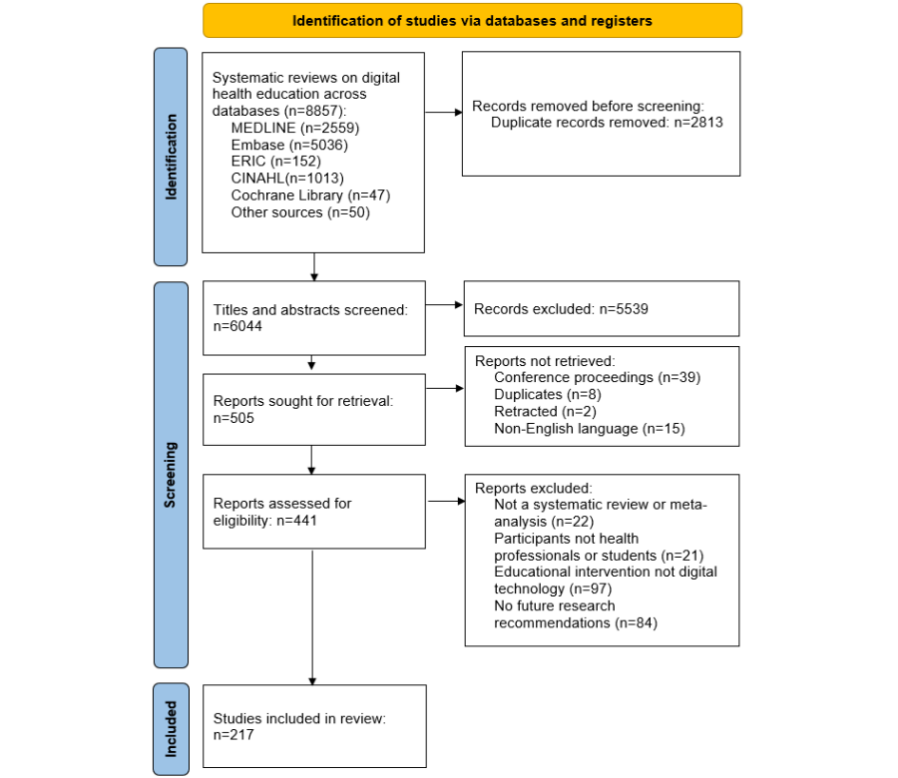
PRISMA (Preferred Reporting Items for Systematic Reviews and Meta-Analyses) flow diagram charting identification of studies.

A total of 8857 potentially relevant papers were identified across all information sources. Following removal of duplicates, of the 8857 papers, 6044 (68.24%) were screened by title and abstract. Of these 6044 papers, 441 (7.3%) were assessed for eligibility via full-text review, leading to 217 (3.6%) papers being included in this review (Figure 1). Refer to Multimedia Appendix 6 for a complete list of key study characteristics for all included studies.

#### Study Characteristics

All included papers were systematic reviews or meta-analyses. Of the included studies across all the systematic reviews, high-income settings were predominant, with 23% (50/217) exclusively consisting of high-income countries and 33.6% (73/217) including a mix of high- and middle-income countries. In total, 14.3% (31/217) covered high-, middle-, and low-income countries, with only 0.9% (2/217) identifying middle- and low-income countries. The remaining 28.1% (61/217) of systematic reviews did not report the economic setting of their included studies.

In total, 29% (63/217) of the systematic reviews were focused on preservice alone. Of these, 11% (24/217) were nursing students, 5.1% (11/217) were medical students, and 7.8% (17/217) were mixed student populations. The least common population groups to be studied in the included reviews were pharmacy students (4/217, 1.8%), dentistry students (4/217, 1.8%), physiotherapy students (2/217, 1.8%), and health sciences students (1/217, 0.5%). This reflects the number of publications from these disciplines.

Reviews exploring the use of interventions with in-service professionals (44/217, 20.3%) are divided into fewer, less distinct categories: 13.8% (30/217) physicians, 6.5% (14/217) mixed health care professionals, and 0.5% (1/217) health care workers.

Of the systematic reviews comprising studies for both pre- and in-service professionals (110/217, 50.7%), 22.1% (48/217) focused on medical students and physicians, 17.5% (38/217) focused on mixed students and health care professionals, and 6% (14/217) focused on nursing students and nurses. Reviews comprising studies on dentistry, pharmacy, and physiotherapy were less common: 1.4% (3/217) dentistry students and dentists, 0.9% (2/217) pharmacy students and pharmacists, and 0.5% (1/217) physiotherapy students and physiotherapists.

The included reviews investigated digital education interventions for learners at a variety of stages in their career: 45.2% (98/217) were created for both students and graduates, 29.5% (64/217) for students only, 10.1% (22/217) for practicing postgraduate health care professionals, 7.8% (17/217) for a mix of trainees and postgraduate health care professionals, and 7.4% (16/217) for graduate trainees.

**Figure 2:**
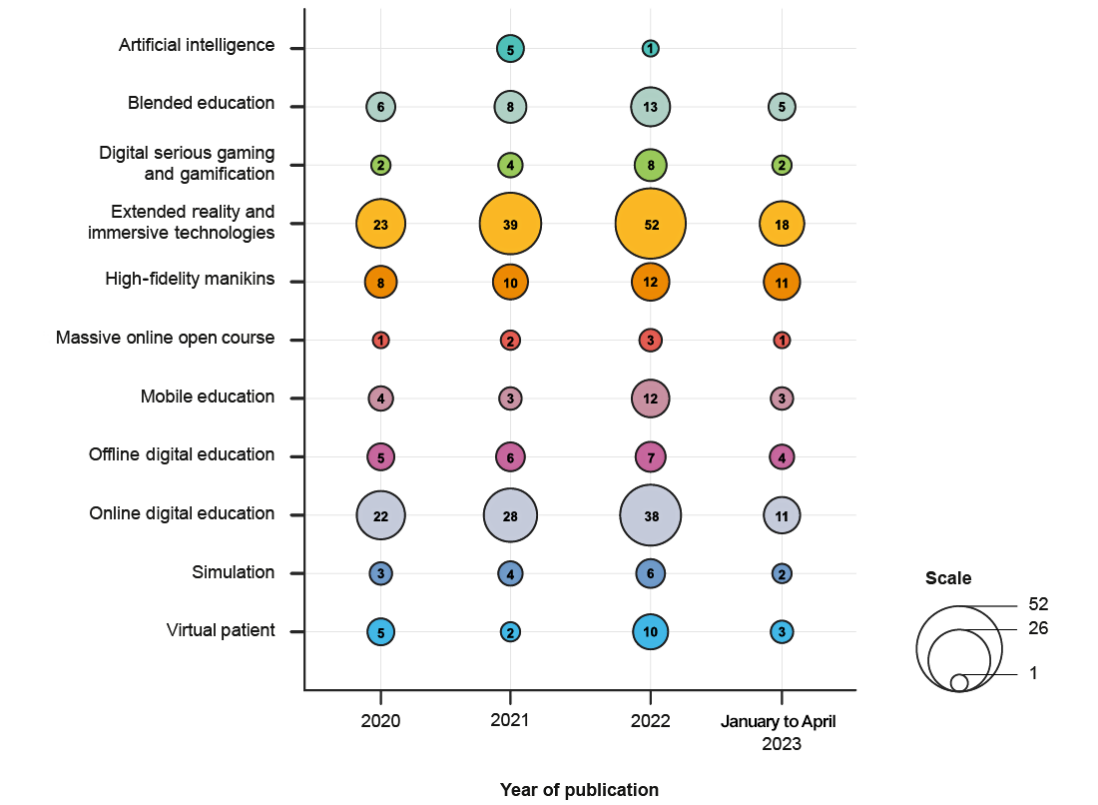
Number of systematic reviews published per year up to April 2023 for each digital modality.

Figure 2 displays a chart showing the number of systematic reviews published for each digital modality, organized by publication year. Several additional digital modalities were identified in the systematic reviews over and above the modalities included in the study by Tudor Car et al [26]. These were consolidated into three new categories:

1. AI: this category includes traditional AI and generative AI, as well as novel AI-driven education modalities such as personalized learning, adaptive learning, AI-enabled extended reality (XR), chatbots, and virtual learning coaches. It also covers AI as an enabling technology, for example, when it helps educators produce content or generates data-driven insights.
2. XR and immersive technologies: this category includes augmented reality, XR, mixed reality, and haptic technology. Virtual reality was also reclassified to fall within this definition.
3. Simulation: this category includes high-fidelity simulators and robotic simulation.

Refer to Multimedia Appendix 2 for a full list of definitions.

Most systematic reviews focused on XR and immersive technologies (132/217, 60.8%) and online digital education (99/217, 45.6%). The least focus was given to serious gaming and gamification (16/217, 7.4%), simulation (15/217, 6.9%), massive online open courses (7/217, 3.2%), and AI (6/217, 2.8%).

Publication numbers were highest in 2022, although the annual totals between January 2020 and April 2023 (Figure 2) exceed those published in the original study by Tudor Car et al [26].

Mapped against the conceptual framework of digital health education for health care professionals [26], most systematic reviews were focused on education modality (170/217, 78.3%) and instructional design (99/217, 45.6%). Very few reviews focused on human resource requirements (7/217, 3.2%) and regulatory factors (6/217, 2.8%) in relation to digital education infrastructure, with only 0.9% (2/217) of reviews concerned with quality assurance of digital health education in the setting.

The majority of studies emphasized reporting educational outcomes related to skills (161/571, 28.1%) and knowledge (133/571, 23.3%), followed by studies focusing on satisfaction (77/571, 13.5%), attitude (54/571, 9.5%), and behavior (38/571, 6.7%). Few studies (29/571, 5.1%) reported patient outcomes.

#### Future Research Recommendations

A total of 151 future research recommendations were extracted from the 217 systematic reviews. These were analyzed, recategorized, and consolidated by 3 authors (AP, CM, and EW) to create a final list of 63 updated research questions. Refer to Tables 2 and 3 for the complete lists.

Across the questions, online digital education (299/1257, 23.7%) and XR and immersive technologies (266/1257, 21.1%) were the most predominant digital modalities requiring further research. Most questions suggest further inquiry within the following domains of education: modality (21 questions), instructional design (16 questions), and assessment (14 questions).

The least predominant modalities are massive online open courses (20/1257, 1.59%) and AI (18/1257, 1.43%).

**Table 2:** New research questions identified from the included papers.

New research questions identified from the included papers	Evidence source references
Can the positive outcomes observed in studies at levels 2B and 3 of the Kirkpatrick model be further validated through studies at level 4, and what are the potential organizational and participant-level changes that may result from implementing these techniques?	[32]
How do immersive technologies impact learning outcomes?	[32-40]
How do we measure the learning transfer from digital education to the clinical setting?	[41-45]
How does research and evidence inform education commissioning and selection of digital technologies?	[46]
How does the design of VPS^a^ impact learning outcomes in health professions education and training curriculum?	[47,48]
How does the use of social media as a pedagogical tool contribute to learner efficacy?	[49,50]
How effective is blended learning in different disciplines, age groups, and learning environments for nursing students?	[51]
How can digital health education be used to offer an inclusive learning experience for staff and students?	[52-55]
How can we optimize the use of AI^b^, machine learning, and deep learning to facilitate education and training?	[56,57]
What are health care students’ learning needs, and can they be met by the use of digital gamification?	[58-64]
What are the advantages and disadvantages of the recognized standard setting methods in simulation-based training?	[58,65-67]
What are the challenges of setting up digital simulation education in primary care?	[68]
What are the experiences and attitudes of teachers and trainers toward developing and implementing digital learning designs in health care education?	[69-71]
What are the outcomes and implications of high-quality randomized controlled trials evaluating the impact of virtual reality and augmented reality simulators on the entire robotic surgery learning curve?	[37,72]
What are the specific effects of video as a learning delivery method?	[73]
What automated performance metrics might prove effective in remote proctoring and assessment? For example, eye tracking	[61]
What is the comparative efficacy of various e-learning platforms in teaching specific subtopics when standardized guidelines for information delivery and assessment are used?	[74,75]
What is the effectiveness of digital health education on long-term retention of skills and knowledge?	[70,74,76-80]
What is the effectiveness of high-fidelity simulation compared with other forms of simulation training?	[81-84]
What is the effectiveness of immersive technologies compared with other forms of simulation training?	[67,80,85]
What is the effectiveness of synchronous digital education compared with asynchronous digital education to deliver health professions education?	[86-88]
What is the impact of digital health education on clinical outcomes in the short and long term?	[89-93]
What is the long-term impact of the COVID-19 pandemic on the educational gaps of health care learners?	[94-97]
What is the validity of using AI, machine learning, and deep learning to generate automated feedback for procedural skills training?	[98,99]

^a^VPS: virtual patient simulation.

^b^AI: artificial intelligence.

**Table 3:** Research questions identified from the included papers and mapped to questions originating from the study by Tudor Car et al [26].

Research questions identified from the included papers	Evidence source references
What are the instructional design barriers and facilitators that affect the continued adoption of digital tools in health professions education?	[63,69,88,100-104]
How does the frequency and duration of digital simulation–based psychomotor skills training affect health professionals’ skills transfer to the clinical setting?	[105-111]
How does the interactivity of digital education programs affect the learning and clinical outcomes of health professionals?	[89,91,105,112-117]
What are the technical resources needed to deliver digital education to health care professionals?	[36,80,105,107,118-122]
Is spacing digital simulation–based training more or as effective as traditional education in clinical psychomotor skills development?	[41,80,105-109,111,117,123,124]
What are learners’ acceptability of digital education with different levels of interactivity?	[87,91,105,107,115,122,125-129]
What is the differential impact of digital education on the clinical performance of trainee or expert surgeons?	[107,120,124-126,130-133]
Can digital education be used to overcome challenges in delivering content-specific topics for health professions education (eg, surgical training in rare pathologic states)?	[33,75,105,107,109,121,125,128,134-137]
Which components of digital health education (eg, interactivity and feedback) contribute to enhanced learning outcomes?	[55,58,61,80,89,115,125,128,129,138-140]
Can digital simulation–based training be used to train nontechnical skills in health professionals?	[32,85,97,111,123,126,128,131,136,141-143]
Is health professions’ digital education more time efficient than traditional education?	[95,105,107,115,123-126,144-149]
Which learning theories can be used to inform the development of effective digital health professions education?	[62,63,69,74,81,87,126,150-157]
Does digital simulation–based psychomotor skills training provide any benefit to medical trainees?	[37,107,115,120,123-125,145,147,158-163]
How does health professions’ digital education affect individual and health services outcomes and organizational practice?	[87,95,105,107,112,123,124,133,134,146,147,158,164-167]
What is the effectiveness of using digital education to train and assess nontechnical skills in health care professionals?	[42,82,84,88,95,111,117,128,131,143,168-170]
How do cost and cost-related outcomes influence the adoption of digital technology in health professions education?	[36,65,107,115,117,123,135,145,167,171-175]
Which features of digital education (eg, technical features, fidelity, safety, and adaptability) affect the learning outcomes of health professions education?	[36,82,104,105,112,122,124-126,129,130,134,139,142,145,171,176,177]
Is mastery learning via digital education more or as effective as traditional education in terms of clinical psychomotor skills improvement?	[34,35,80,105,108,109,115,123,124,134,147,155,158,178-181]
What are health professionals’ attitudes toward digital delivery of education and training programs?	[55,69,70,87,89,91,105,107,112,115,122,125,126,128,146,148,174,182,183]
What are the challenges of digital education for health professionals training in different socioeconomic settings?	[32,68,122,140,171,172,175,184-186]
Can digital education be designed to achieve learning outcomes denoted in the Kirkpatrick model?	[32,40,74,87,107,112,117,124,128,133,134,140,141,147,155,156,164,187,188]
What are health care professionals’ learning needs and can they be met using digital simulation training?	[88,104,105,107,115,117,120,122,123,125,126,128,129,138,146,148,158,162, 164,183,189,190]
What type of instructional design is used in the effective digital education of health professions education?	[33,42,80,82,87,104,117,119,125,126,128,129,135,137,139,156,162,190-194]
What is the optimal duration, frequency, and intensity of digital health professions education programs to improve the learning and clinical outcomes for health care professionals?	[41,69,80,82,102,106-108,110,111,114,124,127,141,161,175,184,186,195]
What is the long-term cost-effectiveness of digital education compared with traditional education for health professionals?	[35-37,48,78,81,85,91,95,105,107,115,120,123,148,159,167,169,173-175,196,197]
How does providing access to digital education improve the learning outcomes of health professionals?	[36,87,89,105,107,109,112,113,115,122-126,129,133,134,138,142,145,147,149,162, 164,167,174,187]
How should learning outcomes in the field of health professions’ digital education be defined and standardized?	[41,45,66,81,87,98,103,104,107,120,121,125,128,129,133,134,141,145,147,149, 156,164,165,174,198-202]
What pedagogy should be used in the digital education of health professionals to improve their knowledge and skills?	[74,78,81-83,87,88,95,102,106,114,117,122,126,150,154-156,165,170,191,192, 201-204]
Can digital education complement (ie, blended) or substitute traditional education for health professionals?	[34,41,43,56,61,64,80,87,91,95,96,104,115,119,124,126,128,140,145,154,156, 187,205-209]
How can digital technology be incorporated into the current health professions education and training curriculum to improve learning outcomes?	[41,80,95,99,105,107,111,112,121,123-127,129,133,134,147,165,168,198, 208,210-214]
What is the ideal approach to assessing health professionals’ knowledge, skills, attitudes, satisfaction, and clinical outcomes from digital technology–based education and training programs?	[32,51,61,83,87,102-104,117,120,123,128,129,133,140,149,156,164,165,176,178, 184,192,193,196,200,201,215-224]
How should studies on digital health professions education be reported?	[37,38,45,46,74,79,82,87,89,91,101,102,104,105,107,114,123,125,126,128,129,134, 140,147,148,153,176,178,184,188,201,202,204,211,223,225-227]
Is digital education effective in improving health professionals’ knowledge and skills performance in the clinical setting?	[36,44,62,87,95,104,105,107,109,113-115,117,123-126,131,133,134,144,145,147,149, 154,158,162,164,176,187,190,192,194,196,209-211,213,215,228]
Which performance metrics or measurement instrument should be used to assess health professionals’ knowledge, skills, attitudes, satisfaction, and clinical outcomes from digital technology–based training programs?	[33,41,46,51,58,61,66,82,87,98,102,104,107,109,120,123,124,128,129,133,140,141, 145,147,153,156,164,166,169,170,178,179,191,199-201,211,215,220,222,229]
How does the design of digital education interventions (eg, format and modality) in health professions education and training curriculum affect learning outcomes?	[33,37,40-42,47,48,54-56,61,63,64,73,80-82,87,95,96,101-103,112,113,119,126, 128,130,131,133,138,142,152,154,196,210,213,230-233]
How should studies of digital health professions education be designed to ensure the generalizability of their findings across different settings?	[38,51,52,62,76,79,80,87,91,92,102,104,105,107,114,115,120,123-126,128,129,133, 134,138,140,141,144,145,147-149,164,170,172,176,184,200-202,205,207,211,223, 225-227,234-238]
What is the impact of digital simulation–based training on clinical outcomes in the short and long term?	[35,37,40,48,68,70,76,81,87,93,106,108,112,117,123,124,128,131-134,140,143, 146,147,155,164,165,174,179,181,185,186,188,196,197,201,218,227,228,236,239-241]
What is the effectiveness of digital education (mixed or single modality) compared with nondigital education to deliver health professions education?	[33,34,36,43,53,56,64,68,75,85,87,89,91,95-97,104,115,119,123,124,126,128,129,131, 134,136,138,140,145,147,149,154,159,161-164,181,192,196,198,205-207,209, 225,230,242,243]
What are the methodological requirements for high-quality, rigorous studies assessing the outcomes of digital health education?	[35,37-39,42,46,47,51,52,57,60,62,63,70,72,74,79-83,86,87,91,100,102,104,105,107, 113-115,123-126,128,129,133,134,140,141,144,145,147-149,152,153,168,176,179, 184,185,188-190,195,199,201-204,214,218,222,223,225-227,234,236,238,241,244-247]

### Delphi Consensus

A total of 42 experts were enrolled to participate in the Delphi consensus activity. Of these, there were 11 academics, 18 clinical educators, 14 simulation faculty, 6 immersive technology professionals, 6 workforce and transformation professionals, 1 learner, and 13 TEL professionals.

In round 1, a total of 151 research questions identified from the literature review were consolidated into 63 questions. These 63 questions were then used to form the web-based survey for round 2.

In round 2, experts assessed each question against the set criteria (scientific merit, significance on workforce development, innovation, relevance to the Long Term Workforce Plan, and feasibility for further study). Two participants chose to withdraw from the Delphi process after reviewing the survey. There was a 78% (31/40) response rate, with nonconsensus thresholds across all questions. Therefore, all 63 questions went through to the next round.

In round 3, in total, 53% (21/40) experts responded to the survey to determine the priority with which each question ought to be pursued with further research. A total of 18 research questions met the consensus threshold for the final round.

In the fourth and final round, the response rate was 55% (22/40), and consensus was reached on the 5 research questions (Table 4) that the expert group identified as the most important and likely to impact the future delivery of digital education.

**Table 4:** Top-rated 5 research questions.

Research question	Sum of score
How do we measure the learning transfer from digital education to the clinical setting?	47
How can we optimize the use of AI^a^, machine learning, and deep learning to facilitate education and training?	38
What are the methodological requirements for high-quality, rigorous studies assessing the outcomes of digital health education?	32
How does the design of digital education interventions (eg, format and modality) in health professions education and training curriculum affect learning outcomes?	31
How should learning outcomes in the field of health professions’ digital education be defined and standardized?	30

^a^AI: artificial intelligence.

## Discussion

### Principal Findings

This study presents findings from 217 systematic reviews on digital education in health care, published between 2020 and 2023. The findings expand upon previous research conducted by Tudor Car et al [26] and define current gaps in the evidence for the efficacy of digital education. After analysis and consolidation, this study identified 63 future research questions: 24 new questions derived from the umbrella review and 39 from the original study by Tudor Car et al [26]. Most reviews focused on XR and immersive technologies as well as web-based digital education, targeting a mixed audience of students and in-service health care professionals. This was also the case for studies with the least commonly represented population groups (dentistry, pharmacy, and physiotherapy).

The 63 research questions were then prioritized through a consensus process with 40 experts from health care and academia, resulting in 5 key research questions (Table 4). These questions span several domains outlined in the conceptual framework [26]: assessment, engagement, learner, level of education, modality, and research.

### The 5 Top-Rated Research Questions

Each of the top-rated questions is supported by a wealth of recommendations for conducting research in digital education. A high-level summary of this evidence is provided for each question.

#### Question 1: How Do We Measure the Learning Transfer from Digital Education to the Clinical Setting?

The evidence behind this research question points to several related yet distinct avenues of inquiry. A common theme among them is the need for further research to demonstrate how learning outcomes translate into real-world contexts, both for technical and nontechnical skills, particularly in nursing and medical fields [42-44]. One study specifically recommends further research to ensure that the competence and confidence in clinical practice skills demonstrated in a learning environment are maintained when applied in the clinical setting, where additional external factors may come into play [44]. Ensuring that the learning experience is relevant and closely mirrors real-world situations is a key consideration when designing instruction to facilitate effective transfer of knowledge and skills [248].

Furthermore, studies should track and evaluate the effectiveness of learning throughout a health care professional’s career [41], using clinical outcomes and patient-reported outcome measures to assess the transfer of learning [45].

#### Question 2: How Can We Optimize the Use of AI, Machine Learning, and Deep Learning to Facilitate Education and Training?

The use of AI to optimize health education is a rapidly developing field. Current evidence proposes further research on the potential of this technology to elevate and shift performance assessment from benchtop and simulated environments to real-world context, offering more precise and scalable feedback mechanisms for both trainees and practitioners. For instance, machine learning has already shown promise in surgical skills training, where it could provide automated, objective assessments of technical skills, provided that more reliable assessment data were available [57]. Besides enhancing existing approaches to learning and assessment, AI also enables new modalities, such as personalized learning, which can enable more self-directed learning and may also support learner motivation by connecting data-driven insights to learning experiences, and adaptive learning [249]. AI integrated into personalized or adaptive learning could help students practice clinical decision-making by simulating different patient cases customized to their knowledge gaps based on previous performance. Furthermore, AI could generate dialogue, actions, and complex situations that replicate clinical settings, further enabling students to develop critical skills with reduced risk [56]. However, while AI offers substantial benefits, its implementation must be carefully considered to mitigate against potential challenges such as ethical concerns, bias from training data, and over-reliance on technology [249]. To safely and effectively adopt AI into health care education, a careful approach could integrate AI in a way that complements existing strategies that emphasize critical thinking and adaptability as well as technical proficiency.

#### Question 3: What Are the Methodological Requirements for High-Quality, Rigorous Studies Assessing the Outcomes of Digital Health Education?

Across the spectrum of digital education modalities, several studies highlighted the need for research to adopt appropriate study designs to determine learning gains [35, 63, 80, 87, 102,123,128,129,179,199,203,226]. Designs need to be larger in scale [39,60,105,107,113,124,134,140,188,214,218,246], multicentered [45,134,227,245], and carry out longer-term follow-up [37,62,81,105,107,128,133,140,149,195,223,241] to ensure retention and transfer of learning. Many of the included studies recommended more randomized controlled trials [37,38,62,81,100,105,115,124,140,188,195,214,222,236,241], although one paper supports the suggestion provided by Tudor Car et al [211] that quasi-experimental designs may be better suited to educational research [144] and another study, in relation to cultural competency, suggests that observational studies and other designs may be preferred [52].

#### Question 4: How Does the Design of Digital Education Interventions (eg, Format and Modality) Used in Health Professions Education and Training Curriculum Affect Learning Outcomes?

Key recommendations from the data sources that derive this research question are primarily concerned with the integration and sequencing of pedagogical devices (ranging from flipped classrooms, timing of simulation briefing, e-learning, and active vs didactic approaches) in health education training and curricula as well as the quality of the approach used [41,42,82,95,102,119,133,142]. Some studies suggest delving further by closely comparing and evaluating the impact of nuanced difference in the design elements of an intervention or specific educational technology [37,63,82,101,130].

Stepping back from this detail within the intervention design itself, studies also reinforce the need to define educational needs, the context, and the setting to select suitable, appropriate, and effective modalities for specific learner groups [40,80,103,126,232].

Looking to the future, some studies highlight several novel technologies and suggest exploring their potential for designing more advanced educational interventions, such as AI [56], adaptive learning [87], wearable technology [213], eye tracking for assessment [55,61], and gamification to increase learner motivation [61].

While many still recommend further comparison studies between digital and nondigital interventions [33,48,119], others support the recommendation by Tudor Car et al [26] that comparisons between digital-to-digital interventions are now needed and necessary [142,231].

#### Question 5: How Should Learning Outcomes in the Field of Health Professions Digital Education Be Defined and Standardized?

Principally, the foundations of this research question lie within a body of evidence that urges a more standardized approach to outcome measurement, using validated measurement tools [81,98,120,141,165]. Beyond this, there is little guidance on how to achieve this, although 1 study (specific to surgical skills training) suggests that machine learning could help identify which metrics accurately assess skills [201].

In many ways, each of these research priorities are connected and interdependent. For example, to be able to measure learning transfer, learning outcomes need to be defined from the beginning and integrated into the learning design. This, in turn, will influence the research methodology used.

### Comparison With Prior Work

This study builds upon and extends research by Tudor Car et al [26]. By doing so, the study not only updates their findings to include data up to 2023 but also extends them by prioritizing 5 research questions through Delphi consensus, thereby addressing a limitation previously identified by Tudor Car et al [26].

In comparison to the 6-year period covered by Tudor Car et al [26], the 3-year timeframe (2020 to 2023) in this study shows a dramatic increase in the number of publications on digital education in health care (from 77 to 217). This surge of activity is reflective of the rise in uptake of digital education in response to the COVID-19 pandemic.

Research with medical students and physicians has seen the greatest increase (from 12% to 22% of studies) and accounts for the largest portion of studies included in this review. In contrast, most earlier studies were more heterogeneous, with a combination of students and health professionals spanning several professions and specialties [26]. Dentistry staff and students remain one of the least represented groups in the literature, along with pharmacy and physiotherapy. The digital education modalities studied align with the broader outcomes of this study (namely, virtual reality, augmented reality, and web-based digital education), and their recommendations for future research mainly focus on the benefits, effectiveness, and perceptions of these modalities.

This study sees the addition of 3 new modalities: AI (in the form of personalized learning, adaptive learning, AI-enabled XR avatars, chatbots, and virtual learning coaches), XR and immersive technologies, and high-fidelity simulation. There has been an explosion of research around the use of XR technologies (132/217, 60.8% of included studies) as well as the emergence of AI (6/217, 3% of included studies). Aside from virtual reality, these technologies were not present in the earlier study.

There was a slight increase (from 5% to 14%) in the number of studies from high-, medium-, and low-income countries [26]. This could be indicative of greater collaborative relationships during the COVID-19 pandemic, as digital education was used globally to overcome the challenges of social distancing in health education delivery. Although evidence relating to digital education research in low-income countries continues to be sparce, the data from this study suggest that future research should focus more closely on learner audiences and intercultural differences to better understand what works across various socioeconomic settings [175,237].

While there have been noticeable shifts in the research populations and the education technologies studies since the findings shared by Tudor Car et al [26], some aspects remain unchanged. Studies comparing digital education interventions with traditional education remain popular, with a continued emphasis on the randomized control trial study design. Comparison studies between digital and nondigital approaches have made significant progress in recent years. Researchers are now encouraged to focus more on comparisons between digital methods in future studies to further advance digital education research. However, despite a move toward research focusing on the context of the learner since the study by Tudor Car et al [26], this review has shown that there continues to be a lack of studies relating to quality assurance, human resources, and regulatory aspects of digital education infrastructure.

### Strengths and Limitations

The findings of this extensive umbrella review replicated a robust methodology used by Tudor Car et al [26] and expanded it to identify the most pressing research questions currently needing attention in digital education within UK health care. Furthermore, these findings were strengthened by consultation with 40 stakeholders across health care and academia to arrive at consensus for the 5 research questions.

By replicating the methodology used by Tudor Car et al [26], this study was constrained by many of the same limitations set out in their study, particularly the exclusion of original research outside of systematic reviews. In addition, this study excluded conference abstracts and non-English papers. However, the impact of excluding non-English papers is considered minimal on the overall outcome [250], and while there may be advantages in including conference abstracts in a systematic review [251], they did not provide the level of information required for data extraction in this study.

There was very low representation from the learner stakeholder group on the Delphi expert panel. Initially, there was uncertainty among the research team members about whether learners would be classed as “experts” for a Delphi panel, which delayed promotion to this group. According to Nasa et al [252], an “expert can be defined as someone with knowledge and experience on a particular subject matter.” In addition, there were similarities drawn with the concept of “experts by experience” [253]. While there are significant differences between being a patient, carer, or service user and a student or learner, the principles of “experts by experience” were considered to be similar. This approach involves gathering the views of people receiving the interventions, in this case, their views and expertise regarding their experience with education. Therefore, a heterogeneous group of stakeholders including final-year undergraduate health care students and postgraduate students or trainees, including doctors in training, was considered applicable to the aims of this study.

### Next Steps

Having defined 5 high-level, key research priorities, it remains to determine what the next steps should be. Stefanidis et al [19] observed that, in the field of surgical simulation, little actual research evidence has been generated despite numerous papers attempting to define priorities. To avoid repeating this shortcoming, the next stage should be to identify specific research questions that have the greatest potential to have significant and practical impact across the system. As proposed by Stefanidis et al [19], establishing a number of expert working groups to devise specific, high-impact and feasible research proposals based on these 5 priorities is suggested. These ideas will subsequently be developed into formal research projects including research questions, collaborators, project design, and required resources. It is anticipated that successful completion of these projects will answer important questions about the practice of digital education in health care.

### Conclusions

This review provides a list of current research gaps in digital education in health care, presenting them as a set of research questions along with the sources from which they are derived.

While the ongoing focus of digital education research on medical trainees and professionals is acknowledged, there is a need for more research involving nursing and allied health professions. It also highlights that there continues to be a need to address the quality assurance, human resources, and regulatory aspects of digital education infrastructure through research.

The prioritized set of 5 research questions, developed with input from expert representatives across health care and academia, is expected to serve as a valuable guide for researchers, funding agencies, and educators. For researchers, these questions offer a helpful starting point for addressing some of the existing gaps in knowledge. For funding agencies, they provide a framework for allocating resources to the areas identified as top priorities for the health care system. Finally, for educators and education providers, these questions can inform decision-making regarding implementation, curriculum design, and quality.

## Appendix

Multimedia Appendix 1 JBI checklist for systematic review and research syntheses.

Multimedia Appendix 2 Definitions of digital education technologies.

Multimedia Appendix 3 Search strategy: Ovid MEDLINE.

Multimedia Appendix 4 Participant recruitment advertisement.

Multimedia Appendix 5 Round 4 preparatory work.

Multimedia Appendix 6 Characteristics of the included systematic reviews.

## Abbreviations

AI: artificial intelligence
NHS: National Health Service
PRISMA: Preferred Reporting Items for Systematic Reviews and Meta-Analyses
XR: extended reality
