# Supplementary material for: Identifying Research Priorities in Digital Education for Health Care: Umbrella Review and Modified Delphi Method Study
Source: J Med Internet Res. 2025 Feb 19;27:e66157. doi: 10.2196/66157 (PMC11888089; doi:10.2196/66157)
Supplement: Multimedia Appendix 1 [file jmir_v27i1e66157_app1.doc]

**Multimedia Appendix 1: JBI Checklist for systematic review and research syntheses**

|  | Yes | No | Unclear | Not applicable |
| --- | --- | --- | --- | --- |
| 1. Is the review question clearly and explicitly stated? | 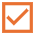 | □ | □ | □ |
| 1. Were the inclusion criteria appropriate for the review question? | 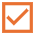 | □ | □ | □ |
| 1. Was the search strategy appropriate? | 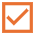 | □ | □ | □ |
| 1. Were the sources and resources used to search for studies adequate? | 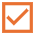 | □ | □ | □ |
| 1. Were the criteria for appraising studies appropriate? | 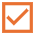 | □ | □ | □ |
| 1. Was critical appraisal conducted by two or more reviewers independently? | 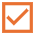 | □ | □ | □ |
| 1. Were there methods to minimize errors in data extraction? | 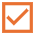 | □ | □ | □ |
| 1. Were the methods used to combine studies appropriate? | 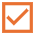 | □ | □ | □ |
| 1. Was the likelihood of publication bias assessed? | □ | 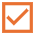 | □ | □ |
| 1. Were recommendations for policy and/or practice supported by the reported data? | 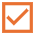 | □ | □ | □ |
| 1. Were the specific directives for new research appropriate? | 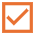 | □ | □ | □ |
