# Supplementary material for: Identifying Research Priorities in Digital Education for Health Care: Umbrella Review and Modified Delphi Method Study
Source: J Med Internet Res. 2025 Feb 19;27:e66157. doi: 10.2196/66157 (PMC11888089; doi:10.2196/66157)
Supplement: Multimedia Appendix 2 [file jmir_v27i1e66157_app2.doc]

**Multimedia Appendix 2: Definitions of digital education technologies**

**On-line digital education:** An online educational programme that is usually delivered via the internet through a learning platform. Gives the learner the opportunity to work at their own pace and to decide when learning takes place. Often referred to as Technology Enhanced Learning.

**Massive open online course (MOOC):** Free online courses to which anyone can enrol. The course takes place entirely online and is delivered by a variety of methods such as videos, quizzes, discussions and reading and research activities. Typical MOOCs last 6-8 weeks

**Mobile education:** Also referred to as mLearning it is method of accessing learning content through mobile devices. This enables learners to access content at the point of need or whenever they want it.

**Serious gaming and gamification:** Use of game structures to teach specific skills, knowledge and attitudes and provide goals beyond entertainment. A good example would be an Escape Room type activity used in a healthcare scenario.

**Extended reality:** Extended reality (XR) is an overarching term that encapsulates current and future developments in augmented reality, mixed reality, and virtual reality

**Augmented reality:** The superimposition of digital data onto the real world, allowing an interaction between the learner, the digital and the physical worlds. Typically done through a smartphone or specific headsets

**Virtual reality (VR):** A simulated experience, created through headsets and advanced software enabling the learner to be completely immersed in a virtual environment. Learners can interact with the environment from a first-person perspective.

**Mixed reality:** A seamless integration of both virtual and digital environments that may be interacted with. A blend of virtual and augmented reality, used in ‘remote assist’ situations.

**Virtual patient:** Two- or three-dimensional avatars on a computer screen or VR headset that help learners engage with a clinical situation or procedure.

**High fidelity simulation:** The use of sophisticated life like manikins that react physiologically as if they were human. Used to create life like situations that allow learners to practice clinical skills without risk of patient harm.

**Blended education:** Also know as hybrid education is a blend of online education, online interaction and traditional classroom-based teaching methods.
