# Supplementary material for: Identifying Research Priorities in Digital Education for Health Care: Umbrella Review and Modified Delphi Method Study
Source: J Med Internet Res. 2025 Feb 19;27:e66157. doi: 10.2196/66157 (PMC11888089; doi:10.2196/66157)
Supplement: Multimedia Appendix 3 [file jmir_v27i1e66157_app3.doc]

**Appendix 3. MEDLINE search strategy**

Search strategy for Medline (Ovid)

1 exp Education, Professional/

2 Education, Veterinary/

3 1 not 2

4 Education, Predental/

5 Education, Premedical/

6 exp Students, Health Occupations/

7 ((medic* or premedic* or dent* or laborator* or predent* or midwi?e* or nurs* or nutrition* or orthop* or podiat* or pharmac* or psycholog* or psychiatr* or health or healthcare or occupational therap* or physiotherap* or physical therap* or clinical or surg* or radiolog* or obstetric* or gyn?ecolog* or orthodont* or An?esthesi* or Dermatolog* or Oncolog* or Rheumatolog* or Neurolog* or Patholog* or P?ediatric* or Cardiolog* or Urolog*) adj3 (student* or graduate* or undergraduate* or staff or personnel or practitioner* or clerk* or fellow* or internship* or residen* or educat* or train* or novice* or tutor*)).kf,tw.

8 3 or 4 or 5 or 6 or 7

9 Computer-Assisted Instruction/

10 exp Internet/

11 Computer Simulation/

12 Patient Simulation/

13 software/

14 Mobile Applications/

15 User-Computer Interface/

16 Video Games/

17 Web Browser/

18 Education, Distance/

19 Computers/

20 exp Microcomputers/

21 exp Cell Phones/

22 Games, Experimental/

23 exp Models, Anatomic/

24 Audiovisual Aids/

25 Educational Technology/

26 Electronic Mail/

27 exp Telemedicine/

28 Telenursing/

29 Telecommunications/

30 Webcasts/

31 exp Videoconferencing/

32 9 or 10 or 11 or 12 or 13 or 14 or 15 or 16 or 17 or 18 or 19 or 20 or 21 or 22 or 23 or 24 or 25 or 26 or 27 or 28 or 29 or 30 or 31

33 ((computer* or digital* or hybrid or blended or "mixed mode" or distance or remote* or electronic or mobile or online* or interactiv* or multimedia or internet or web* or virtual* or game* or gaming or Videogame* or Videogaming) adj3 (classroom* or course* or educat* or instruct* or learn* or lecture* or simulat* or train* or teach* or tutor* or platform*)).kf,tw.

34 (Simulat* adj3 (course* or educat* or instruct* or learn* or train* or teach* or platform* or high-fidelity)).kf,tw.

35 (e-learn* or elearn* or m-learn* or mlearn* or smartphone* or smart-phone* or ((mobile or cell) adj2 phone*) or iphone* or android* or ipad* or Personal digital assistant* or handheld computer* or Mobile App? or Mobile Application? or webcast* or webinar* or flipped classroom* or Serious game* or Serious gaming or Patient Simulat* or Virtual patient*).kf,tw.

36 ((educat* or instruct* or learn* or simulat* or train* or teach* or interactiv*) adj2 technolog*).kf,tw.

37 ("Massive Open Online Course?" or MOOC?).kf,tw.

38 33 or 34 or 35 or 36 or 37

39 (Canvas network or Coursera or Coursesites or edx or Futurelearn or iversity or miriada x or moodle or novoed or openlearning or open2study or plato or spoc or udacity or pingpong).kf,tw.

40 32 or 38 or 39

41 8 and 40

42 Education.fs.

43 Education/

44 Teaching/

45 Learning/

46 Curriculum/

47 exp Inservice Training/

48 "educat*".kf,tw.

49 "learn*".kf,tw.

50 "train*".kf,tw.

51 "instruct*".kf,tw.

52 "teach*".kf,tw. 193227

53 43 or 44 or 45 or 46 or 47 or 48 or 49 or 50 or 51 or 52

54 Health Personnel/

55 exp Allied Health Personnel/

56 Anatomists/

57 "Coroners and Medical Examiners"/

58 exp Dental Staff/

59 exp Dentists/

60 Health Educators/

61 Infection Control Practitioners/

62 Medical Laboratory Personnel/

63 exp Medical Staff/

64 exp Nurses/

65 exp Nursing Staff/

66 Personnel, Hospital/

67 Pharmacists/

68 exp Physicians/

69 Health Occupations/

70 exp Allied Health Occupations/

71 Biomedical Engineering/

72 Chiropractic/

73 exp Dentistry/

74 exp Evidence-Based Practice/

75 exp Nursing/

76 Dietetics/

77 Optometry/

78 Orthoptics/

79 exp Pharmacology/

80 exp Pharmacy/

81 Podiatry/

82 Psychology, Medical/

83 Serology/

84 Specialization/

85 exp Surgical Procedures, Operative/

86 exp Radiography/

87 54 or 55 or 56 or 57 or 58 or 59 or 60 or 61 or 62 or 63 or 64 or 65 or 66 or 67 or 68 or 69 or 70 or 71 or 72 or 73 or 74 or 75 or 76 or 77 or 78 or 79 or 80 or 81 or 82 or 83 or 84 or 85 or 86

88 (Physician* or Doctor* or Nurs* or Surg* or Health Personnel or healthcare professional* or radiolog* or dentist* or Pharmacist* or Hospital Administrator* or Podiatr* or Psycholog* or Psychiatr* or An?esthesi* or Clinician* or Dermatolog* or General practioner* or Cardiolog* or Oncolog* or Rheumatolog* or Neurolog* or Patholog* or P?ediatric* or Physiotherap* or Physical therap* or Occupational therap* or dieti?ian* or Dietetic* or midwi?e* or nutrition* or orthopti* or obstetric* or gyn?ecolog* or orthodont* or Urolog*).kf,tw.

89 87 or 88

90 40 and 53 and 89

91 Motor Skills/

92 ((psychomotor or procedural or technical) adj3 skill*).kf,tw.

93 (psychomotor adj3 performance).kf,tw.

94 91 or 92 or 93

95 8 and 94

96 41 and 90 and 95

97 limit 96 to yr="2020 -Current"

98 ((comprehensive* or integrative or systematic*) adj3 (bibliographic* or review* or literature)).ab,ti.

99 (meta-analy* or metaanaly* or "research synthesis").ab,ti.

100 ((information or data) adj3 synthesis).ab,ti.

101 (data adj2 extract*).ab,ti.

102 98 or 99 or 100 or 101

103 (cochrane adj3 trial*).ab.

104 (cinahl or embase or medline or psyclit or (psycinfo not "psycinfo database") or pubmed or scopus or "sociological abstracts" or "web of science").ab.

105 103 or 104

106 ("cochrane database of systematic reviews" or evidence report technology assessment or evidence report technology assessment summary).jn.

107 "Evidence Report: Technology Assessment*".jn.

108 Meta-Analysis.pt.

109 Meta-Analysis as Topic/

110 (review adj5 (rationale or evidence)).ab,ti.

111 review.pt.

112 110 and 111

113 106 or 107 or 108 or 109 or 112

114 102 or 105 or 113

115 97 and 114
