# Supplementary material for: Identifying Research Priorities in Digital Education for Health Care: Umbrella Review and Modified Delphi Method Study
Source: J Med Internet Res. 2025 Feb 19;27:e66157. doi: 10.2196/66157 (PMC11888089; doi:10.2196/66157)
Supplement: Multimedia Appendix 4 [file jmir_v27i1e66157_app4.doc]

Multimedia Appendix 4: Participant recruitment advertisement

**Call for study participants: Help NHS England Technology Enhanced Learning (TEL) team determine key research priority areas.**

NHS England TEL team is looking for 40 participants to take part in a Delphi study to help determine key research priorities so they can gather evidence and improve how educational technologies are used and supported across health and care.

The team would like to hear from TEL professionals and simulation faculty, clinical educators, health and care workforce development and transformation leads, academics and healthcare students (final year undergraduate) and postgraduate students or trainees, including doctors in training.

This study will help to build on their current research capability with a set of clearly defined priorities that aim to:

- ensure that the techniques and technologies they use are evidence-informed and continue to create the required impact(s).
- stimulate and drive research in the system and contribute new knowledge in healthcare education delivery and workforce development.

The study starts Monday 5^th^ February 2024.

For more information, please contact the study leads [Chris Munsch](mailto:c.munsch@nhs.net) and [Alison Potter](mailto:alison.potter13@nhs.net).
