# Supplementary material for: Identifying Research Priorities in Digital Education for Health Care: Umbrella Review and Modified Delphi Method Study
Source: J Med Internet Res. 2025 Feb 19;27:e66157. doi: 10.2196/66157 (PMC11888089; doi:10.2196/66157)
Supplement: Multimedia Appendix 5 [file jmir_v27i1e66157_app5.doc]

**Multimedia Appendix 5: Delphi round 4 preparatory work**

**Delphi round 4 preparatory work**

Below is a list of the research questions that reached consensus from the previous round of scoring.

Ahead of the round 4 meeting on Wednesday 27^th^ March, please take some time to familiarise yourself with the research questions and consider which five you would consider of top-most priority for further research by NHS England Technology Enhanced Learning. You may wish to make some preparatory notes on these in the column provided.

There will be time within the meeting to share your views on the highest priorities and listen to others’ views before making a final decision at the end of the meeting.

| **QID** | **Research question** | **% rated medium or high priority** | **Select which 5 you consider to be top-most priority** |
| --- | --- | --- | --- |
| 16 | How does the design of virtual patient simulation impact health professions education and training curriculum learning outcomes? | 95% |  |
| 6 | Can digital simulation-based training be used to train nontechnical skills in health professionals? | 90% |  |
| 7 | Does digital simulation-based psychomotor skills training provide any benefit to the medical trainee? | 90% |  |
| 14 | How does research and evidence inform education commissioning, and selection of digital technologies? | 90% |  |
| 22 | How might we optimise use of AI, machine and deep learning to facilitate education and training? | 90% |  |
| 5 | Can digital education complement (ie blended) or substitute traditional education for health professionals? | 86% |  |
| 8 | How can digital technology be incorporated into current health professions’ education and training curriculum to improve learning outcomes? | 86% |  |
| 10 | How do immersive technologies impact learning outcomes? | 86% |  |
| 21 | How might digital health education be used to offer an inclusive learning experience for staff and students? | 86% |  |
| 23 | How should learning outcomes in the field of digital health professions education be defined and standardised? | 86% |  |
| 58 | What is the validity of using AI, machine and deep learning to generate automated feedback for procedural skills training? | 86% |  |
| 9 | How do cost and cost-related outcomes influence the adoption of digital technology in health professions education? | 81% |  |
| 11 | How do we measure the learning transfer from digital education into the clinical setting? | 81% |  |
| 15 | How does the design of digital education interventions (eg format and modality used) in health professions education and training curriculum affect learning outcomes? | 81% |  |
| 17 | How does the frequency and duration of digital simulation–based psychomotor skills training affect health professionals’ skills transfer to the clinical setting? | 81% |  |
| 35 | What are the challenges of digital education for health professionals training in different socioeconomic settings? | 81% |  |
| 36 | What are the challenges of setting up digital simulation education in primary care? | 81% |  |
| 39 | What are the methodological requirements for high-quality, rigorous studies assessing the outcomes of digital health education? | 81% |  |
