# Supplementary material for: Identifying Research Priorities in Digital Education for Health Care: Umbrella Review and Modified Delphi Method Study
Source: J Med Internet Res. 2025 Feb 19;27:e66157. doi: 10.2196/66157 (PMC11888089; doi:10.2196/66157)
Supplement: Multimedia Appendix 6 [file jmir_v27i1e66157_app6.docx]

Multimedia Appendix 6. Characteristics of the included systematic reviews

| **Study ID** | **Study objective** | **Educational outcomes** | **Evidence – GRADE assessment or other scales** | **Setting** | **Framework component** |
| --- | --- | --- | --- | --- | --- |
| Ashokka ​​ et al [1] 2020 | The BEME systematic review was designed to (1) appraise the existing published evidence on educational interventions that are intended for ‘doctors in training’ to teach early recognition and prompt escalation in acute clinical deteriorations (2) to synthesise evidence & to evaluate educational effectiveness. | knowledge, patient outcomes, satisfaction, skills | Other; Buckley et al. 2009; BEME guide No 11). | HIC only | Context (level), Education (modality), Education (instructional design), Education (assessment) |
| Kim​​ et al [2] 2023 | To systematically review and meta-analyze research on the development of XR-based pediatric nursing simulation programs and assess the current status of their development, effectiveness, and limitations. | confidence, knowledge, performance, satisfaction, self-efficacy, skills | Risk of bias, Other; Risk of Bias in Non-randomized Studies of Interventions | HIC only | Context (institutional norms), Context (level), Infrastructure (digital), Education (modality), Education (instructional design) |
| Dromey​ et al [3] 2021 | To investigate the use of high-fidelity simulation in obstetric ultrasound, to identify its usability for learners, and to establish if the skills obtained in a simulated environment can be translated to improved clinical performance. | skills | Medical Education Research Study Quality Instrument (MERSQI); | HIC only | Context (level), Education (modality) |
| De Gagne​​ et al [4] 2023 | This systematic review explored the use of videoconferencing as a teaching tool in response to the pandemic as well as issues related to digital equity and inclusion. | attitude, confidence, knowledge | Medical Education Research Study Quality Instrument (MERSQI); | This systematic review explored the use of videoconferencing as a teaching tool in response to the pandemic as well as issues related to digital equity and inclusion. | This systematic review explored the use of videoconferencing as a teaching tool in response to the pandemic as well as issues related to digital equity and inclusion. |
| Haiser​ et al [5] 2022 | To identify vascular surgery simulation models and assess their validity and levels of effectiveness (LoE) for each model in order to successfully implement them into current training curricula. | attitude, skills, systemic | Other; Messick’s modern concept of validity framework, quantified by Beckman et al. ’s rating scale, adaption of McGaghie’s translational outcomes was applied, and a level of effectiveness score (LoE) from 1 to 5 | Information not available | Context (sociocultural norms), Context (institutional norms), Context (setting), Context (level), Education (modality), Education (instructional design), Education (assessment), Learner, Research |
| Nowell et al [6] 2022 | To conduct a systematic review of the prevalence and diversity of technologies and design strategies used to prepare graduates in caring professions for competent and effective practice in digital working environments. | skills | Other; The Effective Public Health Practice Project Quality Assessment Tool (EPHPP) to rate each quantitative study. And the Joanna Briggs Institute Critical Appraisal Checklist for Qualitative Research. | HIC and MIC | Education (instructional design) |
| Dickinson​​ et al [7] 2020 | The aim of this review was to determine the quality, efficacy, and validity evidence for educational Apps used by surgical residents. | knowledge, attitude, validity | Not reported; | Information not available | Context (sociocultural norms), Context (level), Infrastructure (digital), Education (modality), Education (content), Learner, Research, Quality Assurance |
| Lee​ et al [8] 2020 | To evaluate all simulation models for ophthalmology technical and non-technical skills training and the strength of evidence to support their validity and effectiveness. |  | Not reported, Other; Whilst study quality was not reported, the educational impact of studies was evaluated using Messick’s validity framework and McGaghie’s model of translational outcomes for evaluating effectiveness. | Information not available | Infrastructure (digital) |
| Noyes​ et al [9] 2020 | To synthesise the literature and gather quantitative and thematic data to reveal potential pedagogical limitations and research gaps, as well as to direct future research and provide an evidence-based foundation for future digital credentialing projects in health care education. | attitude, motivation | Medical Education Research Study Quality Instrument (MERSQI), Other; NOS-E (Newcastle-Ottawa Scale-Education) | Information not available | Education (engagement), Education (instructional design) |
| Patel​ et al [10] 2020 | To identify the current simulation-based training models described in the literature, quantitatively assess their validity, and determine their effectiveness for training in neurosurgery. | behavioural, patient outcomes, skills | Other; Messick validity framework | HIC and MIC | Context (level), Education (modality), Education (content), Education (engagement), Education (assessment), Learner |
| Patel​ et al [11] 2020 | The increasing challenges facing the training of future neurosurgeons have led to continued development of simulation-based training, particularly for neurosurgical subspecialties. The simulators must be scientifically validated to fully assess their benefit and determine their educational effects. In this second part, we aim to identify the available simulators for spine, pediatric neurosurgery, interventional neuroradiology, and nontechnical skills, assess their validity, and determine their effectiveness. | behavioural, knowledge, patient outcomes, satisfaction, skills | Other; The modern taxonomy for validity based on Messick was used to assess the studies. Further, a rating scale devised by Beckman et al. was used to evaluate the strength of each source of validity evidence. A level of effectiveness (LoE) was then assigned to each training model using an adapted version of the McGaghie proposed levels of simulation-based translational outcomes | HIC and MIC | Context (institutional norms), Context (setting), Context (level), Infrastructure (digital), Education (modality), Education (content), Education (instructional design) |
| Regmi​​ et al [12] 2020 | Identify and synthesise the factors – enablers and barriers – affecting e-learning in health sciences education (el-HSE) that have been reported in the medical literature |  | Other; ‘QualSyst developed by Kmet and colleagues | HIC and MIC | Context (sociocultural norms), Context (institutional norms), Infrastructure (digital), Education (content) |
| Abualadas​​ et al [13] 2023 | In this systematic review, the educational effectiveness of online anatomy teaching is compared with traditional ("face-to-face”) teaching methods, specifically in terms of students' academic performance and satisfaction. | knowledge, satisfaction, skills | Other; JBI Critical Appraisal Checklists for randomized controlled studies, cross-sectional studies, cohort, and case control studies. | HIC and MIC | Context (level), Education (modality), Education (instructional design) |
| Chan​​ et al [14] 2021 | To assess the ability of these systems to quantitatively determine surgeon performance, compare surgeon performances, or demonstrate progression toward surgical competency. | skills | Not reported; | Information not available | Education (modality) |
| Hippe​​ et al [15] 2020 | to study and characterise existing evidence regarding cost implications, cost-effectiveness, and cost-benefit assessment of simulation-based education, with a particular focus in neonatal resuscitation. | systemic | Not reported; | HIC, MIC, and LIC | Context (institutional norms), Context (sociocultural norms) |
| Moehead​ et al [16] 2020 | To (1) determine the key features that are associated with an effective and functional Web-based education program—an effective and functional Web-based program is defined as one that measures results, is accessible, is user friendly, and translates into clinical practice—and (2) determine how these features correlate with the Dementia Care Competency and Training Network. | behavioural, confidence, knowledge, satisfaction, skills | Other; CASP "system of appraisal" and "Kirkpatrick’s salient elements of the model for the evaluation of training" | HIC only | Context (sociocultural norms), Education (instructional design) |
| Arqub​​ et al [17] 2023 | This review aimed to assess the effectiveness of TEL compared with traditional learning methods in the field of orthodontics. | attitude, behavioural, knowledge, skills | Risk of bias, Other; Cochrane risk of bias tool 2.0 | HIC and MIC | Education (modality) |
| Delungahawatta et [18] 2022 | To examine the scope and impact of e-learning interventions on medical student learning in clinical medicine, in order to aid medical educators when implementing e-learning strategies in programme curricula. | knowledge, satisfaction, skills | Other; Education Group for Guidelines on Evaluation (EGGE) | HIC only | Context (sociocultural norms), Context (institutional norms), Context (setting), Context (level), Infrastructure (digital), Infrastructure (human resources), Education (modality), Education (content), Education (instructional design), Education (engagement), Education (assessment), Learner |
| Sun​ et al [19] 2023 | The aim of this review was to determine the use of VR and AR techniques in hip-related preoperative operation simulator and training, postoperative rehabilitation, and feasibility applications in the operating room. | knowledge, skills | Other; Joanna Briggs Institute Critical Appraisal Checklist Quality Appraisal for Cadaveric Studies scale | Information not available | Context (setting) |
| Hao et al [20] 2022 | To systematically synthesize the available literature on the application of digital education in undergraduate nursing and medical interns during the COVID-19 pandemic | confidence, knowledge, satisfaction, skills | Other; Mixed Methods Appraisal Tool (MMAT) | HIC and MIC | Education (modality) |
| Lakhani​​ et al [21] 2021 | The aim of this article is to review the recent research investigating the use of arthroscopy simulators in training and the teaching of surgical skills. | self-efficacy, skills | Other; Oxford Centre for Evidence-Based Medicine Levels of Evidence guidelines. | Information not available | Education (modality), Education (engagement) |
| Ong et al [22] 2021 | This systematic review aims to identify and investigate the utility of extended reality in ophthalmic education, diagnostics, and therapeutics. | skills | Other; Validity model - Content, Response process, Internal structure, Relationship to other variables, and Consequences, and Oxford Centre for Evidence-Based Medicine (OCEBM) levels of evidence | Information not available | Context (sociocultural norms), Context (institutional norms), Context (level), Infrastructure (digital), Education (modality), Education (content), Education (instructional design), Education (engagement), Education (assessment), Learner, Research |
| Zhang​​ et al [23] 2021 | To critically evaluate research papers that investigated the effectiveness of virtual physiology laboratories for student learning. | knowledge, satisfaction, skills | Other; As the included studies comprised a mixture of both quantitative and qualitative studies, the conventional data analysis tools were not applicable to this review. Therefore, the risk of bias in individual studies was evaluated by using a novel additive scoring system. | HIC and MIC | Infrastructure (digital), Education (modality), Education (instructional design) |
| Chiang​​ et al [24] 2022 | The present study aimed to classify and summarize the application of AR technology in vocational and educational training (VET) and on-the-job training (OJT). | skills | Risk of bias; | HIC and MIC | Context (setting), Context (level) |
| Gelmini et al [25] 2022 | To compare augmented reality (AR) with other learning methods in interventional radiology. | knowledge, skills | Other; Cochrane Collaboration tool, Best Evidence Medical Education (BEME) Collaboration Guide no. 11, Kirkpatrick model (BEME Guide no. 8) | HIC only | Education (modality) |
| Lee​​ et al [26] 2022 | This systematic review aimed to examine authentic learning methods and their effects in healthcare education | knowledge, skills, satisfaction, attitude, behavioural, confidence, wellbeing | Other; Joanna Briggs Institute Checklist for Randomized Controlled Trials and the Checklist for Quasi-Experimental Studies | HIC and MIC | Context (level), Infrastructure (digital), Education (modality), Education (instructional design) |
| Du​ et al [27] 2022 | The primary goal of this analysis is to determine the effectiveness of blended learning versus traditional face-to-face teaching in nursing education from the three aspects of knowledge, skills and satisfaction. | satisfaction, skills | Risk of bias, Other; MINORS (methodological items for non-randomized studies) | HIC and MIC | Context (level), Education (modality) |
| Lockey​​ et al [28] 2022 | This overview of existing systematic reviews aims to evaluate the impact of blended learning on educational outcomes in health care professional education, identify gaps in the current evidence, and direction for future reviews. | attitude, behavioural, knowledge, satisfaction, skills | Grading of Recommendations, Assessment, Development, and Evaluations (GRADE); | HIC, MIC, and LIC | Education (modality) |
| Vallée​​ et al [29] 2020 | The aim of this study was to assess the effectiveness of blended learning compared to that of traditional learning in health education. | knowledge, skills, satisfaction | Risk of bias; | HIC, MIC, and LIC | Context (sociocultural norms), Context (level), Education (modality), Education (instructional design), Learner |
| Kim​​ et al [30] 2023 | To compare online learning with traditional face-to-face and blended learning, based on randomized controlled trials, to determine the impact of online learning on nursing students’ learning outcomes. | attitude, knowledge, satisfaction, skills | Risk of bias; | HIC and MIC | Context (level), Education (modality), Education (instructional design) |
| Lee​​ et al [31] 2021 | This study aims to provide medical educators with insights into the current status and prospects of undergraduate medical education, which has been affected by the COVID-19 pandemic. | systemic | Not reported; | HIC and MIC |  |
| Ahmed​ et al [32] 2020 | To conduct a systematic review of existing literature on simulation-based training of cataract surgery. Available literature was evaluated and projections on how current findings could be applied to cataract surgery training were summarised. | skills | Risk of bias; | Information not available | Context (sociocultural norms), Education (modality) |
| Duarte​​ et al [33] 2022 | To assess whether the effectiveness of distance-learning and/or e-learning, m-learning and web-based methods are equivalent to traditional methods. | behavioural, knowledge, satisfaction, skills | Other; Buckley’s quality indicators | HIC only | Education (modality) |
| Gao​​ et al [34] 2021 | To systematically assess whether MOOCs are superior to the traditional lecture-based method in medical course. | knowledge, satisfaction | Risk of bias, Other; Begg's test | MIC and LIC | Education (modality) |
| Gharib​​ et al [35] 2023 | This systematic narrative review summarises various computer-based simulations described in the pharmacy practice education literature, identifies the currently available products, and highlights key characteristics. | attitude, knowledge, satisfaction, skills | Not reported; | HIC and MIC | Education (modality), Infrastructure (digital) |
| Walshe​​ et al [36] 2022 | To identify strategies used to support culturally authentic experiences and the focus, theoretical framings, and outcomes of cultural simulations. | attitude, behavioural | Medical Education Research Study Quality Instrument (MERSQI); | HIC and MIC | Education (modality), Education (content), Education (instructional design), Education (assessment), Learner |
| Aditya​​ et al [37] 2020 | To examine the current educational interventions utilized to improve the performance of endourology trainees and to critically appraise the strengths and limitations of each. | patient outcomes, skills | Medical Education Research Study Quality Instrument (MERSQI); | Information not available | Context (level), Education (modality), Education (instructional design), Education (engagement), Education (assessment) |
| Hovgaard​​ et al [38] 2021 | We aimed to systematically review the literature on models for simulation-based training and assessment of myringotomy and ventilation tube insertion (MT) and supporting educational evidence. | confidence, knowledge, skills | Risk of bias, Other; Kirkpatrick's hierarchy of educational outcomes Messick’s framework of validity evidence Quality assessment tool developed by Gordon et al. (2018)  Strength of conclusion tool described by Yardley and Dornan (2012) | Information not available | Education (modality), Education (instructional design) |
| O’Connor​ et al [39] 2023 | To identify and synthesise theories that support the design and delivery of digital learning   interventions in nursing and midwifery education. | knowledge, skills | Not reported; | HIC, MIC, and LIC | Education (instructional design) |
| Lo​​ et al [40] 2022 | To examine the challenges to fully online flipped learning and identify useful course-design elements for practicing this instructional approach in health professions education. | knowledge, skills | Not reported; | HIC, MIC, and LIC | Education (modality) |
| Higgins​​ et al [41] 2021 | The aim of this research was to undertake a systematic literature review and investigate the effectiveness of simulation-based medical education interventions for i) increasing transferability of skills acquired from simulated settings to theatre, ii) preventing long-term skills decay or, iii) improve patient-related outcomes. | knowledge, patient outcomes, skills | Medical Education Research Study Quality Instrument (MERSQI); | Information not available | Context (sociocultural norms), Context (level), Education (modality), Education (instructional design), Education (engagement), Education (assessment) |
| Gordon​ et al [42] 2020 | The aim of the current systematic review is to identify the evidence concerning teaching, assessment or other educational developments in response to the COVID-19 pandemic within medical education. Our review will address three main questions:  -What developments or changes in medical education have been deployed? (i.e. description or ‘what was done’ (Cook et al. 2008)).  -What is the impact of these developments or changes? (i.e. evaluation or ‘did it work?’).  -What lessons to be applied in the future have been learned by the teams who deployed these developments or changes? (i.e. implications or ‘what’s next?’). | behavioural, knowledge, satisfaction, skills | Risk of bias, Other; ROBINS-I tool (Risk Of Bias In Non-randomized Studies of Interventions) for non-randomised trials |  |  |
| Jhou​​ et al [43] 2021 | This network meta- analysis aimed to assess the efficacy of different educational methods for healthcare professionals | knowledge, skills | Risk of bias, Grading of Recommendations, Assessment, Development, and Evaluations (GRADE); | HIC and MIC | Context (institutional norms), Infrastructure (human resources), Education (modality), Education (instructional design) |
| Ortega​​ et al [44] 2022 | In this umbrella review, we provide a critical overview of recent systematic reviews examining digital and blended curriculum delivery strategies in graduate health professions education and discuss their implementation in graduate, entry-level physical therapy education programs. | attitude, knowledge, skills | Other; CEP Modified AMSTAR Scale | Information not available | Context (sociocultural norms), Context (institutional norms), Context (setting),Education (modality),Education (content),Education (instructional design),Education (engagement),Education (assessment),Learner |
| Ødegaard​​ et al [45] 2021 | This study aims to identify and investigate the effectiveness of digital learning designs in physiotherapy education. | attitude, knowledge, satisfaction, skills | Risk of bias, Other; Cochrane’s risk of bias tool | HIC and MIC | Education (modality), Education (instructional design) |
| Martinengo​ et al [46] 2020 | To assess the effectiveness of digital education in improving healthcare professionals’ knowledge, attitudes, practical skills and behaviour change on chronic wound management, and their satisfaction with the intervention | attitude, behavioural, knowledge, patient outcomes, satisfaction, skills | Risk of bias; | HIC and MIC | Context (institutional norms), Context (level), Education (modality), Education (assessment) |
| Thangavelu​​ et al [47]2022 | This review aimed to synthesise evidence from experimental studies of the application of digital serious games in developing nursing clinical competence. | attitude, knowledge, skills | Risk of bias, Other; Cochrane's Risk of Bias tool and the Joanna Brigg's Institute Critical Appraisal Tool for Quasi-Experimental Designs. | HIC and MIC | Education (modality) |
| Davies​​ et al [48] 2021 | To determine if extended immersive ward-based simulation programmes improve the preparedness of undergraduate bachelor's degree nursing students to be ward ready for professional practice as a registered nurse. | behavioural, knowledge, patient outcomes, satisfaction, skills, systemic | Joanna Briggs Institute of Meta-Analysis of Statistics Assessment and Review Instrument, Other; Joanna Briggs Institute (JBI) critical appraisal instruments | HIC only | Education (modality), Education (instructional design) |
| Gosak​​ et al [49] 2022 | To review the outcomes of digital tools in behavior change support education.    To assess the outcomes of the use of digital teaching tools and review the assessment instruments to evaluate the research outcomes (e.g., education skills and learning experience) in health and other students following the introduction of digital teaching tools. | confidence, knowledge, skills | Other; Mixed Method Appraisal Tool (MMAT) | Information not available | Education (modality) |
| Lin​ et al [50] 2021 | E-learning is rapidly growing in medical education, overcoming physical, geographic, and time-related barriers to students. This article critically evaluates the existing research on e-learning in plastic surgery | attitude, knowledge | Not reported; | HIC only | Context (sociocultural norms), Context (institutional norms), Context (level), Education (modality), Education (content), Education (engagement), Learner |
| Naciri​​ et al [51] 2021 | The purpose of this review was to explore health science students’ perceptions, acceptance, motivation, and engagement with e-learning during the COVID-19 pandemic. The specific research questions were as follows: (1) What are students’ perceptions of the implementation of e-learning during the COVID-19 pandemic? (2) Did heath profession students accept the adoption of e-learning during COVID-19? (3) What is the motivational level of health professions students towards e-learning during the COVID-19 crisis? (4) What is the engagement level of students during the transition to e-learning during COVID-19? | attitude, satisfaction | Medical Education Research Study Quality Instrument (MERSQI); | HIC, MIC, and LIC | Education (modality), Education (engagement), Learner |
| Aweid​​ et al [52] 2022 | Online journal clubs (JCs) have increased during the COVID-19 pandemic with the resulting social distancing and popularity of online platforms. This systematic review aims to explore current evidence of their use/benefits for clinicians and compare their value to face-to-face (F2F) JCs. | attitude, satisfaction, skills | Medical Education Research Study Quality Instrument (MERSQI); | Information not available | Context (sociocultural norms), Context (level), Education (modality), Education (engagement) |
| Conte​​ et al [53] 2021 | This systematic review aimed to identify the level of impact of educational strategies for teaching tooth carving on the carving ability of undergraduate dental students. | skills | Grading of Recommendations, Assessment, Development, and Evaluations (GRADE), Risk of bias, Other; The Cochrane Risk of Bias Tool 2.0 (Sterne et al., 2019), ROBINS-I tool (Sterne et  al., 2016) | HIC and MIC | Education (modality) |
| Patano​ et al [54] 2021 | To evaluate the effectiveness and teachers/student’s acceptability of e-learning applied to the field of orthodontics and paediatric dentistry. | knowledge, satisfaction | Risk of bias, Other; The risk of bias assessment was performed by a special data form by the software package Review Manager RevMan V 5.1 | HIC and MIC | Context (sociocultural norms), Context (level), Education (modality), Education (instructional design), Education (engagement), Learner |
| Kanika [55] 2020 | The included studies were focusing on the effectiveness of blended learning on nursing students’ knowledge, skills and attitude and the studies that discussed about other methods of teaching and conducted on other specialty students were excluded | attitude, knowledge, satisfaction, skills | Not reported; | HIC and MIC | Context (setting), Context (level), Infrastructure (digital), Education (modality), Education (instructional design), Education (engagement), Learner |
| Asegid​​ et al [56] 2021 | To summarize and produce aggregated evidence on the effect of simulation-based teaching on skill performance in the nursing profession. Simulation is an active learning strategy involving the use of various resources to assimilate the real situation. It enables learners to improve their skills and knowledge in a coordinated environment. | confidence, knowledge, skills | Risk of bias, Joanna Briggs Institute of Meta-Analysis of Statistics Assessment and Review Instrument, Other; Methodological quality was assessed by Joanna Briggs Institute, and the risk of bias was also assessed by Cochrane risk of bias and the risk of bias assessment tool for non-randomized studies (ROBINS-I) checklists. | HIC and MIC | Context (setting), Context (level), Infrastructure (digital), Education (modality), Education (instructional design), Education (engagement) |
| Balakrishnan​​ et al [57] 2021 | The purpose of this study was to evaluate the effectiveness of BL in improving knowledge and skill in pharmacy education. | knowledge, skills | Newcastle-Ottawa Scale (NOS), Other; Modified Newcastle-Ottawa Scale (education) | HIC and MIC | Education (modality), Education (content), Education (assessment), Education (instructional design) |
| Fontaine​​ et al [58] 2021 | The objectives of this study were to identify, appraise, and synthesize the evidence regarding the effect of how media is read i.e. digital vs paper) on reading comprehension in the context of HPE. | knowledge | Risk of bias, Grading of Recommendations, Assessment, Development, and Evaluations (GRADE), Medical Education Research Study Quality Instrument (MERSQI); | HIC only | Infrastructure (digital), Context (sociocultural norms), Education (modality), Learner |
| Özbay​​ et al [59] 2021 | The purpose of this systematic review was to reveal the evidence of how the method of flipped classroom was applied in nursing education and examine the results associated with this teaching method. | attitude, behavioural, knowledge, satisfaction, skills | Other; Quality Assessment Tool for Quantitative Studies (QATQS) | HIC, MIC, and LIC | Context (sociocultural norms), Context (institutional norms), Context (setting), Context (level), Infrastructure (digital), Education (modality), Education (content), Education (instructional design), Education (engagement), Learner |
| Rothschild​​ et al [60] 2021 | Virtual reality simulation (VRS) has become progressively popular as a training tool in ophthalmology. However, debate continues as to whether VRS has resulted in better outcomes for patients after cataract surgery. Accordingly, a qualitative systematic literature review was conducted to identify whether VRS training results in a reduced complication rate after real-life cataract surgery. Included studies measured the effect of VRS on real-life patient outcomes after cataract surgery | patient outcomes, skills | Risk of bias; | Information not available | Context (sociocultural norms), Context (institutional norms), Context (level), Education (modality), Education (instructional design), Education (assessment), Learner, Research |
| Ge​​ et al [61] 2020 | To explore the effectiveness of flipped classroom in radiology education in comparison with traditional didactic or lecture-based pedagogy, with specific indicators focused on students’ objective evaluation or subjective cognition. | knowledge, satisfaction | Newcastle-Ottawa Scale (NOS); | Information not available | Education (modality) |
| Lee​​ et al [62] 2020 | To summarise the design and evaluation of Virtual Patient-based medical communication skills training systems in order to identify features of successful cases. |  | Medical Education Research Study Quality Instrument (MERSQI); Medical Education Research Study Quality Instrument (MERSQI) for quantitative studies, and the QualSyst standard assessment criteria for qualitative studies. | Information not available | Education (instructional design) |
| Keinänen​​ et al [63] 2023 | The objective of this review was to evaluate how effective mentoring education interventions are at improving mentoring competence among health care professionals. | attitude, knowledge, skills | Other; JBI critical appraisal tools for quasi-experimental studies and randomized controlled trials | HIC and MIC | Education (modality), Education (content), Education (instructional design), Education (assessment) |
| Qiao​​ et al [64] 2023 | The three aims identified in this study are (a) to determine whether an effect of NVR simulation exist; (b) to determine whether the effect of NVR simulation is positive or negative; (c) to obtain a single summary estimate of the NVR effect | attitude, confidence, knowledge, self-efficacy, skills | Other; the Cochrane Handbook for Systematic Reviews of Intervention which assisted in evaluating the rigor of the studies. | HIC and MIC | Education (modality) |
| Sezgin​​ et al [65] 2023 | This systematic review and meta-analysis study was conducted to evaluate the effectiveness of interprofessional simulation-based education programs for improving teamwork and communication among students in the healthcare profession. | attitude, knowledge, skills | Risk of bias; | HIC, MIC, and LIC | Context (level), Education (modality), Education (engagement), Education (assessment) |
| Tonapa et al [66] 2023 | The aim of the study was to review and analyze the effectiveness of high-fidelity simulation on learning outcomes in undergraduate nursing education | confidence, knowledge, skills | Risk of bias; | HIC and MIC | Context (sociocultural norms), Context (level), Education (modality), Education (assessment), Learner, Research |
| Baashar et al [67] 2022 | The aim of this work was to assess how effective AR is in training medical students when compared to other educational methods in terms of skills, knowledge, confidence, performance time, and satisfaction | confidence, knowledge, performance, satisfaction, skills | Risk of bias; Cochrane criteria for risk of bias |  |  |
| Min​​ et al [68] 2022 | To systematically summarize research employing serious games in nurse education, to examine their effectiveness, to provide recommendations and implementation strategies, and to suggest future directions for the development and application of serious games in nurse education. | engagement, knowledge, performance, satisfaction, skills | Risk of bias, Other; Cochrane Risk of Bias Tool for Randomized Trials Risk of Bias in Non-Randomized Studies of Interventions (ROBINS-I; Sterne et al., 2016). | HIC only | Education (modality), Education (instructional design) |
| Moussa​​ et al [69] 2021 | The aim of this systematic review was to determine whether virtual technologies have positive effects on dental education outcomes and to explore the attitudes of dental students and educators toward these technologies. | attitude, knowledge, satisfaction, skills | Not reported; | HIC and MIC | Education (instructional design), Education (engagement), Learner |
| Piot​​ et al [70] 2022 | To evaluate the effectiveness of simulation training in psychiatry for nursing students, nurses and nurse practitioners. | attitude, behavioural, knowledge, skills, wellbeing | Risk of bias, Grading of Recommendations, Assessment, Development, and Evaluations (GRADE), Medical Education Research Study Quality Instrument (MERSQI); | HIC, MIC, and LIC | Education (modality) |
| Al Asmri​ et al [71] 2020 | To synthesise evidence regarding the effectiveness of technology-enhanced simulation (TES) for acquiring digital rectal examination skills. | behavioural, confidence, knowledge, patient outcomes, satisfaction, skills, wellbeing | Other; Modified MERSQI | HIC only | Education (modality) |
| Berg​​ et al [72] 2021 | To determine the reported effect of online communication skills training (CST) on health professional (HP) communication skills and patient care outcomes in cancer and palliative care. | attitude, knowledge, patient outcomes, satisfaction, skills | Other; JBI Critical Appraisal Checklists for Randomised Controlled Trials (RCTs), Quasi‐Experimental Studies (non‐randomised experimental studies) and Qualitative Studies | HIC only | Education (modality) |
| Chae​​ et al [73] 2021 | This study aimed to review the effectiveness of virtual simulations and their design features in developing clinical reasoning skills among nurses and nursing students. | patient outcomes, performance, skills | Risk of bias, Grading of Recommendations, Assessment, Development, and Evaluations (GRADE); | HIC and MIC | Learner |
| Woon​​ et al [74] 2021 | The objective was to (1) evaluate the effectiveness of virtual reality (VR) training in improving knowledge among nursing students and (2) identify the essential features of training. | knowledge | Risk of bias, Grading of Recommendations, Assessment, Development, and Evaluations (GRADE); Cochrane Collaboration Risk of Bias | HIC and MIC | Education (modality) |
| Yogeswaran​​ et al [75] 2021 | The study objective was to investigate whether online mindfulness interventions can be utilised  to promote mental health for medical students. | behavioural, wellbeing | Not reported; | HIC only | Learner |
| Berry​​ et al [76] 2020 | The results of TEL effectiveness in periodontics education are controversial. Therefore, this systematic review aimed to evaluate the effectiveness of TEL to improve educational outcomes in the periodontics field compared to traditional learning methods | attitude, behavioural, knowledge, patient outcomes, skills | Risk of bias; | HIC only | Context (sociocultural norms), Context (institutional norms), Context (level), Education (content), Education (modality), Education (instructional design), Education (assessment) |
| Khalaf​​ et al [77] 2020 | To investigate the effectiveness of technology-enhanced teaching and assessment methods of undergraduate preclinical skills in comparison to conventional methods. | satisfaction, skills | Risk of bias, Grading of Recommendations, Assessment, Development, and Evaluations (GRADE),Other; Cochrane risk of bias tool (RoB 2.0) | HIC and MIC | Education (modality) |
| Lapierre​​ et al [78] 2020 | To investigate the effect of interprofessional manikin-based simulation training on teamwork of real teams during trauma resuscitation in civilian, adult emergency departments. | attitude, behavioural, knowledge, skills | Other; Joanna Briggs Institute Critical Appraisal Tools, JBI Critical Appraisal Checklist for Quasi-Experimental Studies | HIC only | Context (setting), Education (modality) |
| O'Brien​​ et al [79] 2023 | The objective of this systematic review was to determine whether simulated interdisciplinary activities in the health care or clinical setting improve interprofessional collaboration within health care teams that include respiratory therapists. | confidence | Other; Qualitative Checklist from the Critical Appraisal Skills Program | HIC and MIC | Context (sociocultural norms), Context (institutional norms), Context (setting), Context (level), Infrastructure (digital), Infrastructure (regulatory), Education (modality), Education (content), Education (instructional design), Education (engagement), Education (assessment), Learner, Research |
| Lei​​ et al [80] 2022 | This meta-analysis was designed to systematically determine the effect of high-fidelity simulation teaching on nursing students’ knowledge level, professional skill level and clinical ability. | knowledge, skills | Other; Cochrane Handbook (5. 1. 0) quality evaluation criteria | HIC, MIC, and LIC | Education (modality) |
| Susilawati​​ et al [81] 2022 | The limited ability of educational institutions to prepare learning materials due to being expensive makes it essential to use other methods such as digital methods or minimize the use of excessive learning materials. Accordingly, the present systematic review aims to find out the effects of multimedia e-books and Augmented Reality (AR) on the knowledge and skills of health sciences students. | knowledge, satisfaction, skills | Risk of bias, Joanna Briggs Institute of Meta-Analysis of Statistics Assessment and Review Instrument; Joanna Briggs Institute (JBI) checklist for randomized clinical trials (Tufanaru et al., 2017), the JBI checklist for cohort studies (Moola et al., 2017), the JBI checklist for (non-randomized) experimental studies (Tufanaru et al., 2017), and the JBI checklist for qualitative research (Lockwood et al., 2015). | HIC and MIC | Context (sociocultural norms), Context (institutional norms), Context (level), Education (modality), Education (engagement), Learner |
| Chen​​ et al [82] 2021 | To assess the effects of mobile learning for nursing students in clinical nursing education. | confidence, knowledge, satisfaction, self-efficacy, skills | Other; Cochrane Handbook for Systematic Reviews of Interventions | HIC and MIC | Education (assessment), Education (modality) |
| Polce​​ et al [83] 2020 | To perform a systematic review and meta-analysis of the effects of training simulators on orthopaedic surgical skill measures across randomized controlled trials. The authors hypothesized that simulated training would (1) result in objective improvements in skill acquisition and (2) be heterogeneousregarding the outcomes and types of validity assessed. | skills | The Jadad scale; | Information not available | Education (modality), Education (instructional design) |
| Woodhead​​ et al [84] 2020 | The aim of this review was to summarise and critically appraise the available evidence for the effects of simulation on gynaecological ultrasound scan training. | attitude, behavioural, engagement, knowledge, patient outcomes, satisfaction, skills | Risk of bias, Newcastle-Ottawa Scale (NOS); | HIC only | Context (sociocultural norms), Context (level), Education (modality), Education (content), Education (instructional design), Education (assessment), Learner |
| Maheu-Cadotte  ​​ et al [85] 2021 | This study aimed to review the effectiveness of virtual simulations and their design features in developing clinical reasoning skills among nurses and nursing students. | knowledge, satisfaction, skills | Risk of bias, Grading of Recommendations, Assessment, Development, and Evaluations (GRADE); | HIC and MIC | Context (sociocultural norms), Context (level), Infrastructure (digital), Education (modality), Education (instructional design), Learner |
| Muirhead​​ et al [86] 2021 | This review aimed to appraise and synthesise contemporary experimental evidence that evaluated technology-enabled dementia education for health and social care practitioners. Outcomes based on Kirkpatrick’s Model were learner satisfaction; knowledge, skills, and attitudes; behaviours; and results. | attitude, behavioural, knowledge, patient outcomes, satisfaction, skills | Medical Education Research Study Quality Instrument (MERSQI), Other; Mixed Methods Appraisal Tool (MMAT) | HIC only | Context (sociocultural norms), Context (institutional norms), Context (level), Infrastructure (digital), Education (modality), Education (instructional design), Education (engagement), Education (assessment), Learner, Research |
| Banks​​ et al [87] 2022 | To investigate the effect of implementing flipped classroom designs on academic performance, student satisfaction and self-efficacy in undergraduate health science disciplines. | knowledge, satisfaction, self-efficacy | Other; standard quality indicator approach (Buckley et al., 2009) | HIC only |  |
| Chawla​​ et al [88] 2022 | Neurosurgical training has been traditionally based on an apprenticeship model. However, restrictions on clinical exposure reduce trainees’ operative experience. Simulation models may allow for a more efficient, feasible, and time-effective acquisition of skills. Our objectives were to use face, content, and construct validity to review the use of simulation models in neurosurgical education. | skills | Medical Education Research Study Quality Instrument (MERSQI); | HIC and MIC | Context (setting), Context (level), Education (modality), Education (instructional design), Education (engagement), Education (assessment) |
| Chen​​ et al [89] 2021 | To evaluate the current evidence and opportunities to integrate novel technologies into modern digitalised robotic training curricula. |  | Not reported; | Information not available | Education (modality) |
| Olexa​​ et al [90] 2023 | To analyze the current status of AR use cases with the goal of envisioning future uses of AR in neurosurgical education | attitude, knowledge, satisfaction, skills | Medical Education Research Study Quality Instrument (MERSQI); | HIC and MIC | Education (modality) |
| Lop​​ et al [91] 2022 | The goal of the present systematic review is to survey and broach the topic of XR in neurosurgery, with a focus on education | knowledge, performance, skills | Newcastle-Ottawa Scale (NOS), Other; Newcastle–Ottawa Scale-Education (NOS-E) | HIC and MIC | Context (setting), Context (level), Education (content), Education (assessment), Education (modality) |
| Esteban et al [92] 2023 | To summarize published evidence on Simulation Tool (ST) validation used for surgery education and training. | validity | Medical Education Research Study Quality Instrument (MERSQI); | Information not available | Education (modality), Education (content), Education (instructional design), Education (engagement), Education (assessment) |
| Youhasan​​ et al [93] 2021 | This systematic review aims to evaluate the empirical evidence and refereed literature pertaining to the development, application and effectiveness of flipped classrooms in reference to undergraduate nursing education. | attitude, knowledge, skills | Not reported, Other; An evidence hierarchy classification model | HIC, MIC, and LIC | Education (modality), Education (instructional design), Education (engagement), Education (assessment) |
| CC06/94  [94] 2020 | to assess the efficacy and provide a deeper understanding of the flipped classroom in nursing education. | knowledge, skills | Other; Cochrane Collaboration's tool | HIC only | Education (modality), Education (instructional design) |
| van Gaalen​​ et al [95] 2021 | The purpose of this systematic review was to provide a comprehensive overview of the use and effectiveness of gamification in health professions education and to add to the existing research on gamification in several ways. | attitude, behavioural, knowledge, patient outcomes, satisfaction, skills | Medical Education Research Study Quality Instrument (MERSQI); | HIC only | Education (modality), Education (instructional design), Education (engagement) |
| Berthold​​ et al [96] 2022 | To conduct a systematic review to determine the efficacy of head-mounted display (HMD) virtual reality (VR) in orthopaedic surgical training. | knowledge, skills | Not reported; | Information not available | Education (modality) |
| Corvetto​​ et al [97] 2023 | The objective of this research was to identify and review studies that have evaluated the impact of simulation-based training on health care professionals during epidemics. | behavioural, knowledge, skills | Not reported; | Information not available | Education (assessment) |
| Li et al [98] 2022 | To explore the effectiveness of high-fidelity simulation (HFS) in undergraduate nursing education. | confidence, knowledge, motivation, satisfaction, skills | Other; Quality Appraisal Checklist – Quantitative Intervention Studies - NICE | HIC, MIC, and LIC | Context (institutional norms), Education (modality), Education (instructional design), Education (engagement) |
| Orejuela​ et al [99] 2022 | To evaluate the effect of simulation training vs traditional hands-on surgical instruction on learner operative skills and patient outcomes in gynecologic surgeries. | patient outcomes, skills | Risk of bias, Grading of Recommendations, Assessment, Development, and Evaluations (GRADE),Other; Cochrane Risk of Bias tool (for )11 RCTs and selected items from the ROBINS-I Tool (for NRCS and prepost studies) | HIC and MIC | Education (modality) |
| Astbury​​ et al [100] 2021 | The aim of this review is to synthesize review evidence of SBE best practice in a broad range of pre-registration healthcare programs and contextualize findings in light of relevant educational theory. |  | Other; the Joanna Briggs Institute Critical Appraisal Checklist for Systematic Reviews and Research Synthesis | Information not available | Context (level), Education (modality), Education (instructional design) |
| Lowe​​ et al [101] 2021 | This systematic review aimed to determine the value of Immersive Technology to teach students ophthalmic skills and whether it can supplement or replace conventional teaching practices. | skills | Risk of bias; | HIC only | Education (modality) |
| Mao​​ et al [102] 2021 | Competency and expert skill are the end goals of training, and an evidence- based approach to appropriate utilization of iVR technology to supplement training programs is required. In this review, we aim to explore the current literature on the application of iVR and determine its efficacy in surgical skills acquisition. | satisfaction, skills | Grading of Recommendations, Assessment, Development, and Evaluations (GRADE), Medical Education Research Study Quality Instrument (MERSQI); Cochrane methodology | HIC and MIC | Context (sociocultural norms), Context (institutional norms), Context (level), Infrastructure (digital), Education (modality), Education (content), Education (instructional design), Education (engagement), Education (assessment), Learner, Research |
| Le Lous​​ et al [103] 2020 | We sought to review the literature and investigate the current level of evidence about the clinical benefits of hybrid simulation training in the field of obstetrics according to the Kirkpatrick Hierarchy. | skills, patient outcomes | Not reported; | Information not available | Education (modality) |
| Au​​ et al [104] 2023 | To determine the effects related to scenario validity and group size in high-fidelity simulation among undergraduate nursing students. | knowledge, skills | Other; Quality Appraisal Checklist – Quantitative Intervention Studies developed by the National Institute of Health and Care Excellence (NICE) | HIC and MIC | Context (institutional norms), Context (level), Infrastructure (digital), Education (modality), Education (instructional design) |
| Adewuyi​​ et al [105] 2022 | The aim of this systematic review was to synthesize evidence on the effect of clinical experiential learning approaches on pre-licensure nursing students’ competence in dementia care and to identify associated challenges. | attitude, knowledge, skills | Other; Joanna Briggs Institute (JBI) Critical Appraisal Checklist for analytical cross-sectional studies   JBI Critical Appraisal Checklist for Qualitative Research Mixed Methods Appraisal Tool (MMAT) | HIC and MIC | Context (level), Education (modality), Education (instructional design) |
| Chan​​ et al [106] 2023 | This meta-ethnography sought to synthesise qualitative literature on candidates' and assessors' experiences of vOSCE to evaluate whether it may have a role in future assessment practices. | attitude | Other; Critical Appraisal Skills Programme (CASP) qualitative research checklist | HIC, MIC, and LIC | Education (assessment) |
| Oliveira​​ et al [107] 2023 | To evaluate the simulation design characteristics that may influence the stress, anxiety and self-confidence of undergraduate nursing students during learning.Design: Systematic review with meta-analysis. | behavioural, wellbeing | Other; Risk of Bias in Non-randomised Studies of Interventions tool (ROBINS-I tool) | HIC and MIC | Education (modality), Education (content), Education (instructional design), Learner |
| Gawronski​​ et al [108] 2022 | High Fidelity Simulations (HFS) are increasingly used to develop Non-Technical Skills (NTS) in healthcare providers, medical and nursing students. Instruments to measure NTS are needed to evaluate the healthcare providers’ (HCPs) performance during HFS. The aim of this systematic review is to describe the domains, items, characteristics and psychometric properties of instruments devised to evaluate the NTS of HCPs during HFS. | behavioural, skills | Grading of Recommendations, Assessment, Development, and Evaluations (GRADE), Other; Consensus-based Standards for the selection of health Measurement Instruments (COSMIN) checklist | HIC and MIC | Education (modality), Education (instructional design), Learner |
| Wirth​​ et al [109] 2021 | This systematic review aims to summarise the existing evidence from evaluation studies on the prevention of patient-on-employee violence and aggression in EDs, where the purpose of the studies was to reduce the frequency of violent incidents, to increase  knowledge, skills, or awareness related to violent incidents, or to help ED staff feel safer  and more at ease. | confidence, knowledge, skills | Other; JBI Critical Appraisal Checklist for Quasi-Experimental Studies; JBI Critical Appraisal Checklist for Prevalence Studies | HIC only | Education (modality) |
| Mao​​ et al [110] 2022 | This systematic review aims to assess the effectiveness of online video-based education compared with standard conventional education in teaching basic surgical skills to surgical trainees and students undergoing medical training. | confidence, knowledge, satisfaction, skills | Risk of bias; | HIC, MIC, and LIC | Context (sociocultural norms), Context (institutional norms), Context (level), Education (modality), Education (instructional design), Education (engagement), Education (assessment), Learner, Research |
| Ryan​​ et al [111] 2022 | To assess the learning outcomes of immersive technologies compared with traditional learning modalities with regard to knowledge and the participants’ learning experience in medical, midwifery, and nursing preclinical university education. | confidence, knowledge, satisfaction, skills | Medical Education Research Study Quality Instrument (MERSQI); | HIC and MIC | Education (modality) |
| Sleiman​​ et al [112] 2022 | This systematic review aims to understand better the translation of laparoscopic psychomotor skills, acquired in dry lab training, into a direct patient benefit in actual clinical practice in the operating room. | patient outcomes, skills | Other; Critical Appraisal Skills Programme CASP Checklists for Randomised Controlled trials | Information not available | Context (level) |
| Xu​​ et al [113] 2021 | To explore the learning experiences of using game-based education in nursing students | attitude, behavioural, knowledge, satisfaction, skills | Other; Mixed Methods Appraisal Tool (MMAT) | HIC and MIC | Education (modality), Education (engagement), Education (instructional design) |
| Lam​​ et al [114] 2022 | The aim of this review is to systematically review the literature concerning ML and surgical performance assessment. The aims are primarily to summarize the major ML techniques used to date in surgical skill assessment and to identify the current challenges and barriers in the field; second to understand what the key sources of data used to develop these tools are and the tasks or procedures that have been assessed; and finally, to understand to what extent ML has been successfully employed to assess surgical performance objectively. Through this systematic review, we aim to define future directions and propose new criteria in this emerging field. | behavioural, skills | Medical Education Research Study Quality Instrument (MERSQI); | HIC and MIC | Education (assessment) |
| Tabatabaeichehr​​ et al [115] 2022 | This review investigated medical students’ satisfaction level with e-learning during the coronavirus disease 2019 (COVID-19) pandemic and its related factors | satisfaction | Other; AXIS appraisal tool for cross sectional studies | HIC, MIC, and LIC | Context (sociocultural norms), Context (setting), Education (assessment), Learner, Infrastructure (regulatory), Education (content) |
| Dedeilia​​ et al [116] 2020 | The aim of this systematic review was to identify the challenges imposed on medical and surgical education by the COVID-19 pandemic, and the proposed innovations enabling the continuation of medical student and resident training. |  | Other; structured and critical synthesis | Information not available | Context (sociocultural norms), Context (level), Context (setting) |
| Chandran​​ et al [117] 2022 | This review evaluates the effectiveness of smartphone applications in improving academic performance and clinical practice among healthcare professionals and students. | knowledge, skills | Risk of bias, Newcastle-Ottawa Scale (NOS); | HIC, MIC, and LIC | Context (setting), Context (level), Education (modality), Education (instructional design), Education (assessment) |
| Margulies​​ et al [118] 2020 | In this era of work-hour restrictions, the paucity of effective and accessible microsurgical training resources has prompted many to turn to personal digital technology devices and online resources as one means of an overall educational approach to the acquisition of microsurgical skills. Indeed, there have been numerous efforts to find ways to use newly developed digital technology for microsurgical training through both passive and interactive means. Thus, the authors aim to provide a systematic review of the existing easily accessible digital microsurgery training resources reported in the literature, available on the internet, and available as smartphone/tablet applications | attitude, satisfaction, skills | Not reported; | Information not available | Context (sociocultural norms), Context (institutional norms), Context (setting), Context (level), Infrastructure (digital), Education (instructional design) |
| Chen​​ et al [119] 2021 | to identify existing microscope-free training models and to compare models in their ability to improve a user's microsurgical skills. | skills | Other; MINORS, and JBI Critical Appraisal Checklist | Information not available | Infrastructure (digital) |
| Shorey​​ et al [120] 2022 | To consolidate the evidence around the experiences of nursing undergraduates and faculty members navigating through remote and online education during the COVID-19 pandemic. | attitude, knowledge | Other; Critical Appraisal Skills Program (CASP) checklist | HIC, MIC, and LIC | Education (modality), Learner |
| Stojan​​ et al [121] 2022 | The aim of this review was to synthesize published reports of developments in UGME in response to the COVID-19 pandemic, focusing on the ‘pivot’ to online learning and de novo developments in remote learning for nonclinical educational activities. We addressed the following:    What novel solutions or developments were deployed as institutions pivoted from face-to-face to remote/online learning? (i.e. description, or ‘what was done’) What was the impact of these changes? What educational (Kirkpatrick’s) outcomes have been reported for these medical education developments? (i.e. justification or ‘did it work?’)    What lessons were learned by the teams who deployed these developments that can guide future practice? (i.e. Implications or ‘what’s next?’) | attitude, knowledge, satisfaction, skills | Medical Education Research Study Quality Instrument (MERSQI), Other; A visual RAG (red, amber, green) ranking system to assess risk of bias (Gordon et al) | HIC, MIC, and LIC | Context (sociocultural norms), Context (institutional norms), Education (modality), Education (instructional design) |
| Castillo-Segura​​ et al [122] 2021 | This SLR aims to provide insights on how IoT can help to automate and make the assessment of surgical technical skills more objective, thus tackling also the problem of the low teacher-student ratio in traditional assessment approaches. | skills | Not reported; | Information not available | Infrastructure (digital), Education (assessment) |
| Anugrahsari​​ et al [123] 2022 | This study investigated the implementation of patient safety in the hospital, the significance of patient safety education for students, and the search for effective practical learning methods | attitude, knowledge, skills | Not reported; | Information not available | Context (institutional norms), Context (level), Education (modality), Education (instructional design), Education (assessment) |
| Chan​​ et al [124] 2022 | This systematic review was conducted to identify and evaluate existing PCP cancer survivorship programs in published literature and answer the following questions: (1) What are the behavioral/learning theories, pedagogy, and/or frameworks used in PCP survivorship education programs? (2) What are the effects of PCP survivorship education programs on outcomes for PCPs (e.g., knowledge, attitude, behaviors) and for cancer survivors (e.g., health and clinical outcomes, self-efficacy)? | behavioural, confidence, knowledge, patient outcomes, satisfaction, skills | Other; NIH “quality assessment tool for before-after (pre-post) studies with no control group”, The mixed methods appraisal tool (MMAT) | HIC only | Education (modality), Education (instructional design), Education (assessment) |
| Schafer​​ et al [125] 2022 | To describe outcomes of SBEs (Simulation-based experiences) related to FPDR (Family presence during resuscitation) and to provide recommendations for future work. | knowledge, performance | Other; Johns Hopkins Level of Evidence and Quality Guide | HIC only | Education (assessment), Education (modality), Research |
| Tudor Car​​ et al [126] 2022 | To determine the choice of outcomes, measurement instruments, and the use of measurement instruments with validity evidence in randomized controlled trials (RCTs) on the effectiveness of VR, AR, and MR in medical student education. | attitude, engagement, knowledge, patient outcomes, satisfaction, skills, wellbeing | Other; Consensus-Based Standards for the Selection of Health Measurement Instruments (COSMIN) | HIC, MIC, and LIC | Context (setting), Context (level), Education (modality), Education (content), Education (engagement), Education (assessment) |
| Okano​​ et al [127] 2021 | To assess whether simulation-based education for vascular access improved the success rate and decreased complication rates compared to traditional education. | patient outcomes | Grading of Recommendations, Assessment, Development, and Evaluations (GRADE), Risk of bias; | Information not available | Education (modality), Education (assessment) |
| Grafton-Clarke  ​​ et al [128] 2022 | This systematic review synthesises published reports of medical educational developments and innovations that pivot to online learning from workplace-based clinical learning in response to the pandemic. The objectives were to synthesise what adaptations/innovation were implemented (description), their impact (justification), and ‘how’ and ‘why’ these were selected (explanation and rationale). | attitude, knowledge, satisfaction, skills | Risk of bias, Other; Quality of study methodology: the Cochrane risk bias tool for randomised controlled trials and the ROBINS-I tool (Risk Of Bias in Non-randomised Studies of Interventions) for non-randomised trials  Quality of study reporting: a visual ranking system was used to report the risk of bias for these five areas: underpinning bias, resource bias, setting bias, educational bias, and content bias (Gordon and Gibbs 2014). | HIC and MIC | Context (sociocultural norms), Context (institutional norms), Education (modality) |
| Oh​​ et al [129] 2022 | Using electrocardiogram (ECG) interpretation as an example of a widely taught diagnostic skill, the authors conducted a systematic review and meta-analysis to demonstrate how research   evidence on instruction in diagnosis can be synthesized to facilitate improvement of educational activities (instructional modalities, instructional methods, and interpretation approaches), guide the content and specificity of such activities, and provide direction for research. | knowledge, skills | Medical Education Research Study Quality Instrument (MERSQI); | Information not available | Context (sociocultural norms), Context (level), Infrastructure (digital), Education (modality), Education (content), Education (instructional design), Education (assessment), Learner, Research |
| Pires​​ [130] 2022 | To carry out a systematic review about the perception of pharmacy students on the e-learning strategies adopted during the COVID-19 pandemic. | attitude, satisfaction | Not reported; | HIC, MIC, and LIC | Learner |
| Baetzner​ et al [131] 2022 | To provide an overview of scientifically evaluated training methods and to examine whether certain methods seem to be particularly effective. | behavioural, confidence, knowledge, performance, self-efficacy | Other; the Joanna Briggs Institute (JBI) critical appraisal checklists for RCTs and quasi‐experimental studies | HIC, MIC, and LIC | Education (modality), Education (instructional design) |
| Savage et al [132] 2022 | The aim of this review was to determine the impact of e-learning versus traditional learning on emergency medicine learning outcomes of medical students and junior doctors |  | Risk of bias, Other; Cochrane Risk of Bias tool, Oxford Centre for Evidence-Based Medicine: Levels of Evidence | Information not available | Education (modality) |
| Legoux​​ et al [133] 2021 | To determine whether simulation of critical emergency procedures promotes long‐term retention of skills in nonsurgical physicians. | skills | Medical Education Research Study Quality Instrument (MERSQI); | Information not available | Education (modality), Education (instructional design), Education (assessment), Learner |
| Tori et al [134] 2022 | The systematic review analyses the state of the art regarding serious games for health-related education, and evaluates the following: game elements, platforms, evaluation methods and requirements analysis methods. | evaluation | Not reported; | Information not available | Context (setting), Infrastructure (digital), Infrastructure (human resources), Education (modality), Education (instructional design), Education (engagement) |
| Nayar​​ et al [135] 2020 | The primary objective of this review was to assess the factors that influence accuracy of self-assessment at technical skills across all surgical specialties. The use of self-assessment from retrospective video playback was used in some studies and may be of benefit in surgical training curricula to enhance learning of technical skills | skills | Newcastle-Ottawa Scale (NOS); | Information not available | Education (assessment) |
| Bray et al [136] 2023 | This systematic review sought to determine which simulation-based training (SBT) approaches are adopted for continuing professional development within primary care and appraise their impact. | behavioural, knowledge, patient outcomes, satisfaction, skills | Other; Mixed Methods Appraisal Tool (v18) | HIC and MIC | Education (assessment), Education (modality) |
| Richard​​ et al [137] 2023 | This study aims to assess the effectiveness, in terms of the main Kirkpatrick criteria, of simulation training in suicide risk assessment and crisis intervention for healthcare professionals and gatekeepers. | attitude, behavioural, knowledge, skills | Risk of bias, Medical Education Research Study Quality Instrument (MERSQI), Other; Risk of Bias tool 2.0 (RoB 2.0; Cochrane) | HIC, MIC, and LIC | Education (instructional design), Education (modality) |
| Chou et al [138] 2022 | This review aims to systematically evaluate the currently available evidence investigating the effectiveness of simulation-based training (SBT) in emergency obstetrics care (EmOC) in Low- and Lower-Middle Income Countries (LMIC). | knowledge,skills | Not reported; | MIC and LIC | Context (setting) |
| Heuer ​​ et al [139] 2022 | To provide an enhanced understanding of how simulation-based training (SBT) is utilized by selected allied health professions (AHPs) functioning in acute care, the potential impact that simulations can have on practice, and how the value derived from them can be maximized. | skills | No intervention; |  | Education (instructional design), |
| Tokas​​ et al [140] 2022 | We investigated the current literature for simulators that could be used as a tool for teaching urologists alone or within the boundaries of a course or a curriculum. | skills | Not reported; | HIC only | Education (modality) |
| Zaed et al [141] 2022 | To identify the currently available simulators including VR simulators for neurovascular surgery and endovascular interventions to assess their validity and determine their effectiveness to suggest widespread applicability in educational curricula. | knowledge, skills | ; | HIC only | Context (sociocultural norms), Context (level), Infrastructure (digital), Education (modality), Education (content), Learner |
| Aydın​​ et al [142] 2021 | To identify current simulation-based training models and to evaluate their validity and effectiveness | skills | Other; Messick's Framework | HIC and MIC | Education (modality) |
| Davids​​ et al [143] 2021 | Our aim was to determine the types of simulators in use, their effectiveness in improving clinical skills, and whether we have reached a point of global acceptance. | knowledge, skills | Risk of bias, Medical Education Research Study Quality Instrument (MERSQI), Other; Cochrane risk of bias tool | HIC only | Education (modality) |
| Lindhard​​ et al [144] 2021 | We performed a systematic review of the effects of simulation-based team training on clinical performance and patient outcome. | patient outcomes | Risk of bias, Newcastle-Ottawa Scale (NOS); | HIC, MIC, and LIC | Education (modality), Education (assessment) |
| Tropea​​ et al [145] 2020 | The aim of this systematic review was to summarise and critically appraise controlled studies on simulation training in non-cancer palliative care for HCWs. | behavioural, confidence, knowledge, self-efficacy, skills | Other; Cochrane Effective Practice and Organisation of Care risk of bias criteria | HIC only | Context (level), Education (modality), Education (instructional design), Education (engagement), Education (assessment), Learner |
| Nielsen​​ et al [146] 2020 | The aim of this systematic preferred reporting items for systematic reviews and meta-analysis (PRISMA) review was to examine whether VR patient-specific rehearsal PsR can improve operator skills and techniques and provide an overview of the published literature to evaluate the benefits of the technique in endovascular procedures. To our knowledge, this is the first review that explores PsR connected to endovascular procedures. | attitude, behavioural, knowledge, patient outcomes, satisfaction, skills | Medical Education Research Study Quality Instrument (MERSQI); | Information not available | Context (sociocultural norms), Context (institutional norms), Education (modality), Education (assessment) |
| Sultana et al [147] 2020 | This systematic review aims to map and analyse the existing literature on simulation-based training for caesarean sections to gain insight into the different types of simulation training models and methods available and their impact on training and clinical outcomes. | knowledge, skills | Not reported; | HIC and MIC | Education (modality) |
| Fouasson-Chailloux et al [148] 2022 | To assess the impact of the use of social media on health students concerning their capacity to acquire knowledge and skills, but also their perception on this new learning approach compared to traditional learning. | attitude, knowledge, skills | Medical Education Research Study Quality Instrument (MERSQI); | Information not available | Education (modality) |
| Pietersen​​ et al [149] 2022 | to examine the use of standard setting methods in the context of simulation-based training of surgical procedures | skills | Grading of Recommendations, Assessment, Development, and Evaluations (GRADE); | HIC only | Education (assessment) |
| Guckian et al [150] 2021 | To synthesise evidence regarding social media interventions in undergraduate medical education, to identify features associated with positive and negative outcomes. | attitude, behavioural, knowledge, satisfaction, skills | Medical Education Research Study Quality Instrument (MERSQI), Other; Standards for Reporting Qualitative Research (SRQR) instrument | HIC, MIC, and LIC | Education (modality) |
| Jacob​​ et al [151] 2020 | To systematically explore relevant published literature to synthesize the current understanding of the factors impacting clinicians’ adoption of mHealth tools, not only from a technological perspective but also from social and organizational perspectives. | knowledge | Other; Critical Appraisal Skills Program tool [CASP Qualitative research checklist] | HIC, MIC, and LIC | Context (sociocultural norms), Context (institutional norms), Infrastructure (digital), Infrastructure (human resources), Education (engagement) |
| Jones et al [152] 2022 | This review aims to systematically explore, evaluate and summarise the range of techno-logical strategies within the literature regarding support of the student learning experience during clinical placements. | knowledge, behavioural | Other; Maxwell's six dimensions of quality | HIC and MIC | Context (sociocultural norms), Infrastructure (digital), Infrastructure (human resources), Education (modality), Education (instructional design), Education (engagement), Learner |
| Rangarajan​​ et al [153] 2020 | In this review, we aim to systematically assess the current evidence for the educational value of haptics in VR surgical simulation | skills | Other; quality assessment tool for quantitative studies as described in the Cochrane Handbook of Systematic Reviews of interventions. | HIC only | Education (modality) |
| Rashidian et al [154] 2020 | To identify and critically appraise the evidence concerning various educational interventions outside operating rooms, which may enhance cognitive knowledge or improve psychomotor and human factor skills in liver surgery. | knowledge, skills | Grading of Recommendations, Assessment, Development, and Evaluations (GRADE), Other; NOS - E (Newcastle-Ottawa Scale for Education) | Information not available | Education (assessment) |
| Hildreth​​ et al [155] 2023 | This systematic review aims to identify and synthesize studies evaluating the comparative effectiveness of technology-enhanced simulation in EM. | knowledge, patient outcomes, satisfaction, skills | Medical Education Research Study Quality Instrument (MERSQI), Newcastle-Ottawa Scale (NOS); | Information not available | Education (instructional design), Education (modality) |
| Santos​​ et al [156] 2022 | This systematic review presents the technological means for teaching and learning about human anatomy developed and applied in medical courses in the last ten years, besides the infrastructure necessary to use them. |  | Not reported; | HIC and MIC | Education (modality) |
| Jin et al [157] 2021 | The aims of this study are 1. To evaluate the learning curve of laparoscopic training applying virtual reality compared to no training, box trainers, video trainers, and to traditional trainers.  2. To assess whether VR training could improve the initial stage of learning curve in actual surgery compared to other training methods.  3. To compare which level of trainees benefits more from VR training, novices or intermediates? | knowledge, performance, skills | Risk of bias, Grading of Recommendations, Assessment, Development, and Evaluations (GRADE), Other; Cochrane Rick of Bias Tool; | Information not available | Context (level), Education (modality) |
| Neubauer et al [158] 2023 | to provide a general overview of the current state of Musculoskeletal Ultrasound education worldwide, regarding target groups, teaching staff, didactic methods and course formats, also considering previous problems and future opportunities, to support the continuous improvement of the teaching of Musculoskeletal Ultrasound. | Knowledge, skills | Not reported; | Information not available | Context (sociocultural norms), Context (institutional norms), Context (level), Infrastructure (digital), Infrastructure (regulatory), Infrastructure (human resources), Education (modality), Education (content), Education (instructional design), Education (assessment), Learner |
| Muirhead et al [159] 2021 | To establish the technological and pedagogical characteristics associated with effective technology-enabled dementia education for health and social care practitioners. | knowledge, skills, satisfaction, attitude, behavioural, performance | Other; Mixed Methods Appraisal Tool | HIC and MIC | Education (instructional design) |
| Ozdemir et al [160] 2022 | This mixed-methods systematic review was conducted to examine the effectiveness of High-Fidelity Simulation (HFS) methods on teaching urinary catheterization skills to nursing students. | knowledge, skills, satisfaction | Joanna Briggs Institute of Meta-Analysis of Statistics Assessment and Review Instrument; et al [Randomised and non-randomised experimental studies] Joanna Briggs Institute (JBI) Critical Appraisal Checklist for Randomized Controlled Trials and Quasi-Experimental   Studies.     et al [Mixed methods studies] JBI Critical Appraisal Checklist for Randomized Controlled Trials, or Quasi-Experimental Studies, or Qualitative Research | HIC only | Context (sociocultural norms), Context (level), Infrastructure (digital), Education (modality), Education (content), Education (engagement), Learner |
| Brown et al [161] 2021 | The purpose of this study was to investigate the effect of High-Fidelity Simulation based learning using computerized mannequins on clinical performance and preparedness of physical therapy students and physical therapists in acute cardiorespiratory physical therapy | attitude, behavioural, confidence, self-efficacy | Medical Education Research Study Quality Instrument (MERSQI), Other; Quality Assessment for Qualitative Research Reports (QAQRR) | HIC only |  |
| Helming et al [162] 2021 | To evaluate the content quality of YouTube videos intended for professional medical education based on quality rating tool (QRT) scores and determine if video characteristics, engagement metrics, or author type are associated with quality. | validity | Risk of bias, Other; the Journal of the American Medical Association benchmark criteria (JAMA) score, DISCERN instrument score, Global Quality Score (GQS) | Information not available | Infrastructure (regulatory), Education (content), Education (instructional design), Quality Assurance |
| Fadzilah​​ et al [163] 2021 | A systematic review was conducted on the effectiveness of educational intervention designed to improve primary health-care service providers’ (PHSPs) knowledge, attitude, and practice in managing elder abuse and neglect (EAN) cases. | attitude, knowledge, skills | Risk of bias, Other; Cochrane Collaboration Modified Tool for randomized controlled studies and the Risk Of Bias In Nonrandomized Studies of Interventions tool (ROBINS-I) for quasi-experimental studies (Sterne et al., 2016). | HIC only | Education (modality) |
| Sinacori et al [164] 2021 | The aim of the study was to determine the effect of distance learning on knowledge acquisition in undergraduate second-degree nursing students, a growing population of nursing students. | knowledge, performance | Risk of bias, Other; Cochrane Collaboration’s tool for assessing risk of bias in a systematic review | HIC only | Education (modality), Education (instructional design) |
| Svellingen et al [165]2021 | To examine the use and effects of multiple simulations in nursing education. | confidence, knowledge, skills | Risk of bias, Other; Cochrane Risk of Bias tool; Critical Appraisal Skills Programme (CASP) | HIC only | Education (instructional design) |
| Taba et al [166] 2021 | To analyze the effects of using virtual reality simulations on the development of laparoscopic skills in medical students and physicians. | skills | Other; Cochrane Back Review Group (CBRG) | HIC only | Education (modality) |
| Zhao et al [167] 2021 | To compare the examination pass rate of medical students educated using VR and those receiving traditional education to evaluate the teaching effect of VR in medical education. | knowledge | Newcastle-Ottawa Scale (NOS); | HIC only | Education (modality) |
| Alshehri et al [168] 2023 | The objective of this systematic review is to synthesise findings from current literature about the effectiveness of high-fidelity simulation on the development of clinical reasoning-related skills in undergraduate nurses. | skills | Joanna Briggs Institute of Meta-Analysis of Statistics Assessment and Review Instrument; | HIC, MIC, and LIC | Context (sociocultural norms), Context (setting), Infrastructure (digital), Education (modality), Education (instructional design), Education (engagement), Learner |
| Zhao et al [169] 2020 | This research aimed to examine the general efficiency of VR for teaching medical anatomy. | satisfaction | Risk of bias; | HIC and MIC | Education (modality) |
| Jallad et al [170] 2022 | to evaluate the effectiveness of virtual reality simulation as a teaching / learning strategy on the acquisition of clinical skills and performance, self-confidence, satisfaction and anxiety level in nursing education. | attitude, knowledge, satisfaction, skills | Not reported; | Information not available | Education (modality) |
| Plackett et al [171] 2022 | The aim of this study was to synthesise the evidence to understand the effectiveness of virtual patient tools aimed at improving undergraduate medical students’ clinical reasoning skills. | patient outcomes, behavioural | Medical Education Research Study Quality Instrument (MERSQI), Newcastle-Ottawa Scale (NOS); | HIC, MIC, and LIC | Education (modality), Education (instructional design) |
| Yucel et al [172] 2020 | The aim of this systematic review is to identify and summarize evidence of the effectiveness of simulation-based team training in obstetric emergencies for improving team members' technical skills by using Kirkpatrick's training evaluation model. | skills, satisfaction | Other; Mixed Methods Appraisal Tool (MMAT) Version 2011 | HIC, MIC, and LIC | Education (modality) |
| Capitani et al [173] 2023 | Surgical training is crucial for orthopedics residents during their educational careers. Residents who follow classic training courses are less skilled than those trained with simulators. Virtual simulators are reported to be global learning tools for knee arthroscopy. The primary purpose of our study is to evaluate the current state of use of arthroscopic knee simulators and their actual effectiveness in transfer the skills learned in training to the operating theatre. The secondary purpose is to evaluate if the virtual simulators are better than the others in improve arthroscopic skills. | skills | Not reported; | Information not available | Context (sociocultural norms), Context (level), Infrastructure (digital), Education (modality), Education (instructional design), Education (assessment), Research |
| Gomaa et al [174] 2023 | This systematic review aimed to systematically appraise the currently available evidence regarding endovascular high-fidelity simulation interventions, to describe the over-arching strategies used, the learning outcomes addressed, the choice of assessment methodology, and the impact of education on learner performance. | knowledge, skills, | Other; The reporting quality assessment involved 12 items, which included 3 ‘‘yes/no’’ items reporting on the study design, learner characteristics, and matching of outcomes to objectives. | HIC only | Context (institutional norms), Context (level), Education (modality), Education (instructional design), Education (engagement), Education (assessment) |
| Howlader​​ et al [175] 2022 | Oral and maxillofacial surgery (OMS) teaching is set to undergo a paradigm shift towards competency-based training. With increasing focus on resident skill development and patient safety, computerized simulators are likely to play a more mainstream role in OMS training. A systematic review of the available literature was conducted, in accordance with the PRISMA guidelines, to highlight the scope  of computerized simulation in OMS teaching | skills, patient outcomes, | Other; PRISMA | HIC and MIC | Context (sociocultural norms), Context (level), Infrastructure (regulatory), Education (modality), Education (instructional design), Education (engagement), Education (assessment), Learner |
| Suresh et al [176] 2023 | This review aims to provide an update on the role of augmented reality (AR) in surgical training and investigate whether the use of AR improves performance measures compared to traditional approaches in surgical trainees. | skills, satisfaction, | Not reported; | Information not available | Education (modality), Education (instructional design) context (level) |
| Santos et al [177] 2021 | From March through September of 2020, this systematic review attempted to elucidate experiences, benefits, and challenges enforced in dental  education due to the pandemic, the learning technologies, and methods used to  maintain education. | knowledge, skills, satisfaction | Joanna Briggs Institute of Meta-Analysis of Statistics Assessment and Review Instrument, Other; Joanna Briggs Institute JBI Critical Appraisal Checklist for Case Reports |  | Context (sociocultural norms), Context (setting), Infrastructure (digital), Infrastructure (regulatory), Infrastructure (human resources), Education (modality), Education (instructional design), Education (engagement), Education (assessment), Learner |
| van Schaijik​​ et al [178] 2021 | To explore the current uses and to examine the impact of Twitter on dental education, and to analyze and predict potential models of Twitter for future application in dental training, education, and teaching. | knowledge, satisfaction | Medical Education Research Study Quality Instrument (MERSQI); | HIC only | Education (modality) |
| Srinivasa et al [179] 2020 | Online videos are commonly used in medical education. The aim of this review was to investigate the role of online instructional videos in teaching procedural skills to postgraduate  medical learners. | attitude, knowledge, skills | Other; Winona State University hierarchy of evidence scale, which ranged from levels I–VII of evidence depending on the study type and methodology | Information not available | Context (level), Infrastructure (digital), Education (instructional design), Education (engagement), Learner |
| Almutairi et al [180] 2022 | This systematic review was undertaken to investigate the impact of social media use in relation to student engagement in nursing education. | engagement | Other; Mixed Methods Appraisal Tool (MMAT, version 2018 | HIC only | Education (engagement) |
| Shorey et al [181] 2021 | This review explores the use of the two variations of virtual worlds (dVRS and iVRS) as teaching tools among the nursing population, which consists of nursing students and registered nurses, on any outcomes.    This review has three research questions:        1) How effective is the use of virtual worlds as a teaching tool compared to other teaching methods among the nursing population of nursing students and registered nurses?      2) What are the outcomes that were examined in the included studies using virtual worlds?      3) What are the advantages and disadvantages of using virtual worlds in nursing education and clinical practice? | knowledge, satisfaction, skills | Other; Joanna Briggs Institute's appraisal checklists. The Checklist for Quasi-Experimental Studies (non-randomised experimental studies) was used to appraise quasi-experimental studies, and the Checklist for Randomised Controlled Trials was used to appraise studies that adopted the randomised controlled trial design | HIC and MIC |  |
| Chahal et al [182] 2023 | This review aims to assess the transfer of open and laparoscopic psychomotor skills (gained through surgical simulation or operative experience) to the robot, evaluating their impact on the robotic learning curve. | skills | Risk of bias, Other; Cochrane risk-of-bias tool for randomised trials (RoB2) and the Risk of Bias In Non-Randomised Studies of Interventions (ROBINS-I) tool | Information not available | Context (institutional norms), Education (modality), Education (instructional design), Education (engagement) |
| Fahl​​ et al [183] 2023 | To confirm the effectiveness of spaced training when using a VR-simulator and identify an optimal spacing interval to acquire new surgical psychomotor skills. | performance, skills | Risk of bias, Medical Education Research Study Quality Instrument (MERSQI); | HIC only | Education (instructional design) |
| O'Connor et al [184] 2022 | Synthesise literature on theories that inform technology enhanced learning in nursing and midwifery education. | attitude, confidence, engagement, knowledge, satisfaction, self-efficacy, skills | Other; Not quality appraised | HIC and MIC | Education (instructional design) |
| Jujo​​ et al [185] 2021 | aimed to assess the learning effects of novice transesophageal echocardiography (TEE) simulator training and to identify gaps in existing studies. | knowledge, satisfaction, skills | Risk of bias; | HIC only | Context (sociocultural norms), Context (institutional norms), Context (level), Education (modality), Education (instructional design), Education (assessment) |
| Smith et al [186] 2021 | Correct optical diagnosis of colorectal polyps is crucial to implement a resect and discard strategy. Training methods have been proposed to reach recommended optical diagnosis thresholds. The aim of our study was to present a systematic review and meta-analysis on optical diagnosis training. | knowledge, patient outcomes, skills | Risk of bias, Grading of Recommendations, Assessment, Development, and Evaluations (GRADE); | Information not available | Context (sociocultural norms), Context (institutional norms), Context (level), Education (modality), Education (engagement) |
| Engberg​​ et al [187] 2020 | To report and evaluate research in training and assessment of competence in Resuscitative endovascular balloon occlusion of the aorta (REBOA) and femoral arterial access with the aim to investigate the effect of simulation-based training in the procedure and to provide suggestions for the future design of training programs and assessment tools. | attitude, behavioural, knowledge, patient outcomes, satisfaction, skills | Grading of Recommendations, Assessment, Development, and Evaluations (GRADE), Medical Education Research Study Quality Instrument (MERSQI); | Information not available | Education (modality), Research |
| Cant​​ et al [188] 2023 | The aim of this review was to summarize evidence from reviews of literature to determine the use and effectiveness of virtual simulations in contemporary nursing student education | confidence, knowledge, satisfaction, self-efficacy, skills | Other; JBI Critical Appraisal Checklist for Systematic Reviews Syntheses and Research | Information not available | Education (modality), Context (sociocultural norms), Context (setting), Context (level), Education (instructional design) |
| Stone et al [189] 2020 | This review examines the current evidence of the effectiveness of the use of video or video podcast technology produced either commercially or in-house in developing nursing students' confidence in clinical skills for practice. | knowledge, skills | Other; Mixed Methods Appraisal Tool (MMAT) tool for quantitative, qualitative, and mixed methods studies. | HIC and MIC | Education (assessment), Education (modality), Context (level), Infrastructure (digital), Education (instructional design) |
| Ayivi-Vinz et al [190] 2022 | To review the use of the CPD-REACTION questionnaire, which measures the impact of CPD activities on health professionals’ intentions to change clinical behaviour. We examined CPD activity characteristics, ranges of intention, mean scores, score distributions, and psychometric properties. | attitude, behavioural, knowledge, skills | Other; Mixed Methods Appraisal Tool (MMAT) | HIC, MIC, and LIC | Education (assessment) |
| ShahAli et al [191] 2023 | To assess the effectiveness of e-learning methods in comparison with traditional learning on physiotherapy students’ knowledge, skills and satisfaction/attitude. | attitude, knowledge, satisfaction, skills | Grading of Recommendations, Assessment, Development, and Evaluations (GRADE), Other; EPOC Risk of Bias tool | HIC and MIC | Context (level), Education (modality), Education (instructional design) |
| Verville et al [192] 2021 | To describe the best evidence on the effectiveness of technology-based learning tools designed to improve knowledge of health care providers about clinical practice guidelines (CPGs). | knowledge | Other; Scottish Intercollegiate Guidelines Network checklists for RCTs as well as the National Institutes of Health Checklist for pre- and postintervention trials. | Information not available | Context (level), Education (modality), Education (instructional design), Learner |
| Garnier​​ et al [193] 2023 | This study’s objectives are to provide an overview of simulation’s current place in the field of hospital pharmaceutical technology education, to classify these uses, and to discuss how simulation technologies could be better used in the future. | knowledge, skills | Not reported; | HIC only | Education (modality) |
| Singh et al [194] 2020 | A systematic review was conducted to identify and summarize research on the use of technology to train clinicians in evidence-based treatments (EBTs). | behavioural, knowledge, skills | Risk of bias, Other; Risk of bias was assessed with the National Heart, Lung, and Blood Institute Quality Assessment Tool for Controlled Intervention Studies | HIC and MIC | Context (sociocultural norms), Education (assessment), Education (instructional design) |
| Daniel et al [195] 2023 | The objective of this study is to compare surgical residents receiving and not receiving VBC in terms of technical surgical skill. | satisfaction, skills | Risk of bias; | Information not available | Context (level), Education (instructional design), Learner |
| Tursø-Finnich​​ et al [196] 2023 | The purpose of this review is to investigate the use of VR head-mounted displays (VR-HMD) in medical education and assess how they compare with other learning technologies. | knowledge, skills | Medical Education Research Study Quality Instrument (MERSQI); | Information not available | Education (modality) |
| Beshir​ et al [197] 2022 | To summarise evidence on Virtual Patient Simulation (VPS) use in pharmacy education and the effect of adapting VPS on pharmacy students’ engagement, confidence, knowledge, skills, and satisfaction. | attitude, confidence, knowledge, satisfaction, skills | Medical Education Research Study Quality Instrument (MERSQI); | HIC and MIC | Education (modality) |
| Dzyuba et al [198] 2022 | To scope the evidence for current applications of VR/AR in dental education and to anticipate the potential uses of these technologies in this field. | confidence, knowledge, performance, satisfaction, skills | Risk of bias; | Information not available | Infrastructure (digital) |
| Lucena-Anton et al [199] 2022 | The aim of this review was to analyse the use of virtual and augmented reality (VR/AR) compared to traditional methods for teaching physiotherapy. | performance, satisfaction | Joanna Briggs Institute of Meta-Analysis of Statistics Assessment and Review Instrument, Other; Cochrane Collaboration’s RoB Tool 2.0, The JBI Critical Appraisal Checklist for Quasi-Experimental Studies, the JBI Checklist for Cohort Studies, JBI Checklist for Randomised Controlled Trials, risk of bias in non-randomised studies of interventions (ROBINS-I) tool | HIC and MIC | Education (modality) |
| Umphrey et al [200] 2022 | To synthesize recent virtual global health education activities for graduate medical trainees, document gaps in the literature, suggest future study, and inform best practice recommendations for global health educators. | knowledge, satisfaction, skills | ; | HIC, MIC, and LIC | Education (modality), Education (instructional design), Education (content), Education (instructional design) |
| Gelmini​​ et al [201] 2021 | The aim of this study was to compare virtual reality simulation with other methods of teaching interventional radiology. | skills | Risk of bias, Other; The Cochrane Collaboration tool, BEME Guide No. 11(18) and the Kirkpatrick model of training evaluation described in BEME Guide No. 8 by Steinert et al | Information not available | Education (modality) |
| Kovoor et al [202] 2021 | The aim of this study was to perform a systematic review of the published literature to evaluate the validity and effectiveness of augmented reality in surgical education, and to compare it with other simulation modalities. | skills | Risk of bias, Other; Joanna Briggs Institute (JBI) critical appraisal checklist | Information not available | Education (modality) |
| Moro et al [203] 2021 | The objective of this systematic review and meta-analysis is to evaluate the impact of virtual reality or augmented reality on knowledge acquisition for students studying pre-clinical physiology and anatomy. | knowledge | Risk of bias, Other; Cochrane Collaboration's Risk of Bias tool | HIC and MIC | Education (modality) |
| Lohre et al [204] 2020 | We sought to determine the current evidence of the use of virtual (VR), augmented (AR), and mixed (MR) reality simulators in minimally invasive spine surgery (MISS) and spinal endoscopic surgery, including study quality, level of evidence (LoE), and outcomes. | knowledge, skills | Medical Education Research Study Quality Instrument (MERSQI), Other; modified Oxford Centre for Evidence-Based Medicine (OCEBM) | HIC and MIC | Education (modality) |
| Richardson et al [205] 2020 | The objective was to undertake a systematic narrative review to establish and evaluate virtual patient (VP) use in pharmacy. This included VPs that were used to develop or contribute to communication or counselling skills in pharmacy undergraduates, pre-registration pharmacists and qualified pharmacists | confidence, knowledge, satisfaction, skills | Other; The critical appraisal skills programme (CASP) tools for qualitative research, cohort studies and randomised controlled trials | HIC only | Education (modality), Education (assessment) |
| Asadzandi​​ et al  [206] 2022 | The aim of this systematized review was to determine the factors enhancing metacognitive skills in nursing students. | knowledge, skills | Other; CASP Checklist | Information not available | Education (modality), Education (instructional design), Learner |
| Brown et al [207] 2023 | To summarize the current use of VR or AR for simulation training of healthcare providers  in Disaster Medicine education. | skills, satisfaction, confidence | Not reported; | HIC and MIC | Education (modality) |
| Clerkin et al [208] 2022 | The aim of this systematic review (SR) is to establish if video is a beneficial teaching method when teaching psychomotor skills in nursing. | confidence, knowledge, motivation, satisfaction, skills | Other; Quality appraisal was performed by using RevMan software Version 5.3 (Cochrane Collaboration, 2014) to assess the internal validity of all eight studies. | HIC and MIC | Education (modality), Context (level), Education (instructional design) |
| Sim et al [209] 2022 | This review aimed to evaluate the effectiveness of virtual simulations and their associated design features for developing clinical reasoning skills among nurses and nursing students.  The review was guided by the following research questions:  1. What is the effectiveness of virtual simulation on clinical reasoning in nursing education?  2. What are the essential features in designing virtual simulation that improves clinical reasoning in nursing? | knowledge, skills | Grading of Recommendations, Assessment, Development, and Evaluations (GRADE), Other; Cochrane Collaboration’s tool | HIC and MIC | Education (modality), Education (instructional design) |
| Clarke [210] 2021 | This study aims to conduct a systematic review of relevant literature and analyse the efficacy of VR simulation in orthopaedic surgical training, with a focus on outcomes in comparison to current standard training methods. The question this paper will be asking is does training in VR lead to a greater positive effect on outcomes that reflects real surgical competence, compared to standard training currently used in the orthopaedic curriculum, for surgical trainees of all levels. | skills | Risk of bias, Other; CASP criteria for critical appraisal and Robvis for risk of bias | HIC only | Context (institutional norms), Context (level), Infrastructure (digital), Education (modality), Education (content), Education (instructional design), Education (engagement), Education (assessment), Learner, |
| Lin et al [211] 2021 | To assess the impact of VR training for cataract surgery on the operating performance of postgraduate ophthalmology trainees, measured by operating time, intraoperative complications, postoperative complications, supervising physician ratings, and VR simulator task ratings. | performance | Risk of bias; | HIC and MIC | Education (modality), Education (instructional design) |
| Plotzky et al [212] 2021 | Simulation-based learning is widely used in nurse education, including virtual reality (VR) methods which have experienced a major growth lately. Virtual reality offers risk free and contactless learning. Currently, little is known about what topics of nursing are adopted for VR simulations and how their design meets various educational goals. This review aims to scope existing articles on educational VR nursing simulations, and to analyse approaches from didactic and technical perspectives. | behavioural, knowledge, satisfaction, skills | Other; guidelines for evaluating papers on education interventions (1991) | HIC only | Context (sociocultural norms), Context (institutional norms), Context (level), Infrastructure (digital), Education (modality), Education (content), Education (engagement), Education (assessment), Learner |
| Schmidt et al [213] 2021 | to identify evidence on transferability of surgical skills acquired on robotic VR simulators to the operating room and the predictive value of robotic VR simulator performance for intraoperative performance | skills, patient outcomes | Medical Education Research Study Quality Instrument (MERSQI), Other; modified Newcastle–Ottawa Scale for Education (NOS-E) | HIC and MIC | Education (assessment), Education (modality) |
| Tolarba et al [214] 2021 | The goal of this paper is to evaluate the effectiveness of virtual simulation in nursing education by conducting a systematic review of the literature. | attitude, knowledge, satisfaction, skills | Other; Joanna Briggs Institute (JBI) Critical Appraisal Tools. | HIC and MIC | Education (modality) |
| Foronda et al [215] 2020 | The objective of this review was to identify how virtual simulation impacts nursing student learning outcomes. | attitude, confidence, knowledge, satisfaction, skills | Risk of bias, Other; Melnyk and Fineout-Overholt's (2015) levels of evidence and Critical Appraisal Skills  Programme (CASP) guidelines for bias | HIC and MIC | Context (institutional norms), Context (level), Infrastructure (digital), Education (modality), Education (instructional design), Education (engagement), Education (assessment) |
| Portelli et al [216] 2020 | This meta-analysis had two aims:   comparing virtual reality training with apprenticeship training to determine whether it replaces or supplements the latter and providing data to develop conclusions about the benefits achieved by adding virtual reality training routinely for surgical trainees. | knowledge, skills | Not reported; | Information not available | Context (sociocultural norms), Context (institutional norms), Context (setting), Context (level), Infrastructure (digital), Education (modality), Education (instructional design), Education (engagement), Learner |
| Lee​​ et al [217] 2020 | This study aims to examine the effect of the intervention development of a web-based trauma education program for mental health professionals, focusing on previous domestic and international studies, and to find out the composition of the education program through a systematic literature review. This will be used as basic data to prepare the development direction of a web-based trauma education program for mental health professionals in the future. | confidence, knowledge, performance, satisfaction | Risk of bias; | HIC only | Context (level), Education (modality) |

1. Ashokka B, Dong C, Law LS, et al.,. A BEME systematic review of teaching interventions to equip medical students and residents in early recognition and prompt escalation of acute clinical deteriorations: BEME Guide No. 62. Med Teach 2020; 42(7):724–737. PMID: 32493155.

2. Kim EJ, Lim JY, Kim GM,. A systematic review and meta-analysis of studies on extended reality-based pediatric nursing simulation program development. Child Health Nurs Res 2023; 29(1):24–36. PMID: 36760110.

3. Dromey BP, Peebles DM, Stoyanov DV,. A systematic review and meta-analysis of the use of high-fidelity simulation in obstetric ultrasound. Simul Healthc 2021; 16(1):52–59. PMID: 32675735.

4. De Gagne JC, Koppel PD, Wang EJ, et al.,. A systematic review of videoconferencing in health professions education: the digital divide revisited in the COVID-19 era. Int J Nurs Educ 2023; 20(1):20220068. PMID: 36608319.

5. Haiser A, Aydin A, Kunduzi B, et al.,. A systematic review of simulation-based training in vascular surgery. J Surg Res 2022; 279:409–419. PMID: 35839575.

6. Nowell L, Dhingra S, Carless-Kane S, et al.,. A systematic review of online education initiatives to develop students remote caring skills and practices. Med Educ Online 2022; 27(1):2088049. PMID: 35694798.

7. Dickinson KJ, Bass BL,. A systematic review of educational mobile-applications (apps) for surgery residents: simulation and beyond. J Surg Educ 2020; 77(5):1244–1256. PMID: 32307244.

8. Lee R, Raison N, Lau WY, et al.,. A systematic review of simulation-based training tools for technical and non-technical skills in ophthalmology. Eye 2020; 34(10):1737–1759. PMID: 32203241.

9. Noyes JA, Welch PM, Johnson JW, et al.,. A systematic review of digital badges in health care education. Med Educ 2020 Jul; 54(7):600–615. PMID: 31971267.

10. Patel EA, Aydin A, Cearns M, et al.,. A systematic review of simulation-based training in neurosurgery, part 1: cranial neurosurgery. World Neurosurg 2020 Jan; 133:e850–e873. PMID: 31541755.

11. Patel EA, Aydin A, Cearns M, et al.,. A systematic review of simulation-based training in neurosurgery, part 2: spinal and pediatric surgery, neurointerventional radiology, and nontechnical skills. World Neurosurg 2020; 133:e874–e892. PMID: 31541754.

12. Regmi K, Jones L,. A systematic review of the factors – enablers and barriers – affecting e-learning in health sciences education. BMC Med Educ 2020; 20(1):91. PMID: 32228560.

13. Abualadas HM, Xu L,. Achievement of learning outcomes in non-traditional (online) versus traditional (face-to-face) anatomy teaching in medical schools: a mixed method systematic review. Clin Anat 2023; 36(1):50–76. PMID: 35969356.

14. Chan J, Pangal DJ, Cardinal T, et al.,. A systematic review of virtual reality for the assessment of technical skills in neurosurgery. Neurosurg Focus 2021; 51(2):E15. PMID: 34333472.

15. Hippe DS, Umoren RA, McGee A, et al.,. A targeted systematic review of cost analyses for implementation of simulation-based education in healthcare. SAGE Open Med 2020; 8:2050312120913451. PMID: 32231781.

16. Moehead A, DeSouza K, Walsh K, et al.,. A web-based dementia education program and its application to an Australian web-based dementia care competency and training network: integrative systematic review. J Med Internet Res 2020 Jan 22; 22(1):e16808. PMID: 32012077.

17. Arqub SA, Waleed M, Al-Abedalla K, et al.,. Insight on the influence of technology-enhanced learning in orthodontics' education: a systematic review. Eur J Dent Educ 2023; 27(3):729–745. PMID: 36250284.

18. Delungahawatta T, Dunne SS, Hyde S, et al.,. Advances in e-learning in undergraduate clinical medicine: a systematic review. BMC Med Educ 2022; 22(1):711. PMID: 36207721.

19. Sun P, Zhao Y, Men J, et al.,. Application of virtual and augmented reality technology in hip surgery: systematic review. J Med Internet Res 2023 Mar 10; 25:e37599. PMID: 36651587.

20. Hao X, Peng X, Ding X, et al.,. Application of digital education in undergraduate nursing and medical interns during the COVID-19 pandemic: a systematic review. Nurse Educ Today 2022; 108:105183. PMID: 34741918.

21. Lakhani S, Selim OA, Saeed MZ,. Arthroscopic simulation: the future of surgical training: a systematic review. JBJS Reviews 2021; 9(3):e20.00076. PMID: 33750750.

22. Ong CW, Tan MCJ, Lam M, et al.,. Applications of extended reality in ophthalmology: systematic review. J Med Internet Res 2021 Aug 19; 23(8):e24152. PMID: 34420929.

23. Zhang X, Al-Mekhled D, Choate J,. Are virtual physiology laboratories effective for student learning? A systematic review. Adv Physiol Educ 2021; 45(3):467–480. PMID: 34142876.

24. Chiang F, Shang X, Qiao L,. Augmented reality in vocational training: a systematic review of research and applications. Comput Hum Behav 2022; 129:107125.

25. Gelmini AYP, Duarte ML, Silva MOd, et al.,. Augmented reality in interventional radiology education: a systematic review of randomized controlled trials. Sao Paulo Med J 2022; 140(4):604–614. PMID: 35946678.

26. Lee J, Campbell S, Choi M, et al.,. Authentic learning in healthcare education: a systematic review. Nurse Educ Today 2022; 119:105596. PMID: 36283196.

27. Du L, Zhao L, Xu T, et al.,. Blended learning vs traditional teaching: the potential of a novel teaching strategy in nursing education - a systematic review and meta-analysis. Nurse Educ Pract 2022; 63:103354. PMID: 35580368.

28. Lockey A, Bland A, Stephenson J, et al.,. Blended learning in health care education: an overview and overarching meta-analysis of systematic reviews. J Contin Educ Health Prof 2022 Oct 1; 42(4):256–264. PMID: 36070399.

29. Vallée A, Blacher J, Cariou A, et al.,. Blended learning compared to traditional learning in medical education: systematic review and meta-analysis. J Med Internet Res 2020 Aug 10; 22(8):e16504. PMID: 32773378.

30. Kim JY, Kim M,. Can online learning be a reliable alternative to nursing students' learning during a pandemic? A systematic review and meta-analysis. Nurse Educ Today 2023; 122:105710. PMID: 36739810.

31. Lee IR, Kim HW, Lee Y, et al.,. Changes in undergraduate medical education due to COVID-19: a systematic review. Eur Rev Med Pharmacol Sci 2021 Jun; 25(12):4426–4434. PMID: 34227080.

32. Ahmed TM, Hussain B, Siddiqui MAR,. Can simulators be applied to improve cataract surgery training: a systematic review. BMJ Open Ophth 2020; 5(1):e000488. PMID: 32953997.

33. Duarte ML, Santos LRD, Iared W, et al.,. Comparison of ultrasonography learning between distance teaching and traditional methodology. An educational systematic review. Sao Paulo Med J 2022; 140(6):806–817. PMID: 36043680.

34. Gao J, Yang L, Zou J, et al.,. Comparison of the influence of massive open online courses and traditional teaching methods in medical education in China: A meta-analysis. Biochem Mol Biol Educ 2021; 49(4):639–651. PMID: 33894023.

35. Gharib AM, Bindoff IK, Peterson GM, et al.,. Computer-based simulators in pharmacy practice education: a systematic narrative review. Pharmacy 2023; 11(1) PMID: 36649018.

36. Walshe N, Condon C, Gonzales RA, et al.,. Cultural simulations, authenticity, focus, and outcomes: a systematic review of the healthcare literature. Clin Simul Nurs 2022; 71:65–81.

37. Aditya I, Kwong JCC, Canil T, et al.,. Current educational interventions for improving technical skills of urology trainees in endourological procedures: a systematic review. J Endourol 2020 Jul; 34(7):723–731. PMID: 31691593.

38. Hovgaard LH, Al-Shahrestani F, Andersen SAW,. Current evidence for simulation-based training and assessment of myringotomy and ventilation tube insertion: a systematic review. Otol Neurotol 2021; 42(9):e1188–e1196. PMID: 34267097.

39. O’Connor S, Wang Y, Cooke S, et al.,. Designing and delivering digital learning (e-Learning) interventions in nursing and midwifery education: a systematic review of theories. Nurse Educ Pract 2023; 69:103635. PMID: 37060735.

40. Lo CK, Hew KF,. Design principles for fully online flipped learning in health professions education: a systematic review of research during the COVID-19 pandemic. BMC Med Educ 2022; 22(1):720. PMID: 36229820.

41. Higgins M, Madan C, Patel R,. Development and decay of procedural skills in surgery: a systematic review of the effectiveness of simulation-based medical education interventions. Surgeon 2021 Aug; 19(4):e67–e77. PMID: 32868158.

42. Gordon M, Patricio M, Horne L, et al.,. Developments in medical education in response to the COVID-19 pandemic: a rapid BEME systematic review: BEME Guide No. 63. Med Teach 2020 Nov; 42(11):1202–1215. PMID: 32847456.

43. Jhou H, Ou-Yang L, Lin M, et al.,. Different pedagogies for acquisition of knowledge and skill: a systematic review and network meta-analysis. Postgrad Med J 2021; 98(1162):604–609. PMID: 33931555.

44. Ortega MAC, Marchese VG, Zarro MJ, et al.,. Digital and blended curriculum delivery in health professions education: an umbrella review with implications for Doctor of Physical Therapy education programs. Phys Ther Rev 2022; 27(1):4–24.

45. Ødegaard NB, Myrhaug HT, Dahl-Michelsen T, et al.,. Digital learning designs in physiotherapy education: a systematic review and meta-analysis. BMC Med Educ 2021; 21(1):48. PMID: 33441140.

46. Martinengo L, Yeo NJY, Markandran KD, et al.,. Digital health professions education on chronic wound management: a systematic review. Int J Nurs Stud 2020 Apr; 104:103512. PMID: 32086027.

47. Thangavelu DP, Tan AJQ, Cant R, et al.,. Digital serious games in developing nursing clinical competence: a systematic review and meta-analysis. Nurse Educ Today 2022 Jun; 113:105357. PMID: 35429749.

48. Davies H, Sundin D, Robinson S, et al.,. Does participation in extended immersive ward-based simulation improve the preparedness of undergraduate bachelor’s degree nursing students to be ready for clinical practice as a registered nurse? An integrative literature review. J Clin Nurs 2021; 30(19-20):2897–2911. PMID: 33870592.

49. Gosak L, Štiglic G, Budler LC, et al.,. Digital tools in behavior change support education in health and other students: a systematic review. Healthcare (Basel)&nbsp; 2022; 10(1):1. PMID: 35052165.

50. Lin IC, Lee A, Mauch JT,. Does e-learning improve plastic surgery education? A systematic review of asynchronous resources. Ann Plast Surg 2021 Jul 1; 87(1s Suppl 1):S40–S51. PMID: 34180865.

51. Naciri A, Radid M, Kharbach A, et al.,. E-learning in health professions education during the COVID-19 pandemic: a systematic review. J Educ Eval Health Prof 2021; 18:27. PMID: 34710319.

52. Aweid B, Haider Z, Wehbe M, et al.,. Educational benefits of the online journal club: a systematic review. Med Teach 2022 Jan; 44(1):57–62. PMID: 34403291.

53. Conte DB, Zancanaro M, Guollo A, et al.,. Educational interventions to improve dental anatomy carving ability of dental students: a systematic review. Anat Sci Educ 2021 Jan; 14(1):99–109. PMID: 32717118.

54. Patano A, Cirulli N, Beretta M, et al.,. Education technology in orthodontics and paediatric dentistry during the COVID-19 pandemic: a systematic review. Int J Environ Res Public Health 2021; 18(11):6056. PMID: 34199882.

55. Kanika HK,. Effect of blended learning on academic achievements and attitude of nursing students: a systematic review. &nbsp;Indian J Forensic Med Toxicol 2020; 14(3):383–387.

56. Asegid A, Assefa N,. Effect of simulation-based teaching on nursing skill performance: a systematic review and meta-analysis. Front Nurs 2021; 8(3):193–208.

57. Balakrishnan A, Puthean S, Satheesh G, et al.,. Effectiveness of blended learning in pharmacy education: a systematic review and meta-analysis. PLoS One 2021 Jun 17; 16(6):e0252461. PMID: 34138880.

58. Fontaine G, Zagury-Orly I, Maheu-Cadotte M, et al.,. A meta-analysis of the effect of paper versus digital reading on reading comprehension in health professional education. Am J Pharm Educ 2021 Nov; 85(10):8525. PMID: 34301544.

59. Özbay Ö, Çınar S,. Effectiveness of flipped classroom teaching models in nursing education: a systematic review. Nurse Educ Today 2021 Jul; 102:104922. PMID: 33940481.

60. Rothschild P, Richardson A, Beltz J, et al.,. Effect of virtual reality simulation training on real-life cataract surgery complications: systematic literature review. J Cataract Refract Surg 2021 Mar 1; 47(3):400–406. PMID: 32675654.

61. Ge L, Chen Y, Yan C, et al.,. Effectiveness of flipped classroom vs traditional lectures in radiology education: a meta-analysis. Medicine (Baltimore) 2020 Oct 2; 99(40):e22430. PMID: 33019421.

62. Lee J, Kim H, Kim KH, et al.,. Effective virtual patient simulators for medical communication training: a systematic review. Med Educ 2020 Sep; 54(9):786–795. PMID: 32162355.

63. Keinänen A, Lähdesmäki R, Juntunen J, et al.,. Effectiveness of mentoring education on health care professionals´ mentoring competence: a systematic review. Nurse Educ Today 2023; 121:105709. PMID: 36638727.

64. Qiao J, Huang C, Liu Q, et al.,. Effectiveness of non-immersive virtual reality simulation in learning knowledge and skills for nursing students: meta-analysis. Clin Simul Nurs 2023; 76:26–38.

65. Sezgin MG, Bektas H,. Effectiveness of interprofessional simulation-based education programs to improve teamwork and communication for students in the healthcare profession: a systematic review and meta-analysis of randomized controlled trials. Nurse Educ Today 2023 Jan; 120:105619. PMID: 36343420.

66. Tonapa SI, Mulyadi M, Ho KHM, et al.,. Effectiveness of using high-fidelity simulation on learning outcomes in undergraduate nursing education: systematic review and meta-analysis. Eur Rev Med Pharmacol Sci 2023 Jan; 27(2):444–458. PMID: 36734697.

67. Baashar Y, Alkawsi G, Ahmad WNW, et al.,. Effectiveness of using augmented reality for training in the medical professions: meta-analysis. JMIR Serious Games 2022 Jul 5; 10(3):e32715. PMID: 35787488.

68. Min A, Min H, Kim S,. Effectiveness of serious games in nurse education: a systematic review. Nurse Educ Today 2022; 108:105178. PMID: 34717098.

69. Moussa R, Alghazaly A, Althagafi N, et al.,. Effectiveness of virtual reality and interactive simulators on dental education outcomes: systematic review. Eur J Dent 2021; 16(01):14–31. PMID: 34428851.

70. Piot M, Dechartres A, Attoe C, et al.,. Effectiveness of simulation in psychiatry for nursing students, nurses and nurse practitioners: a systematic review and meta-analysis. J Adv Nurs 2022 Feb; 78(2):332–347. PMID: 34378236.

71. Al Asmri MA, Ennis J, Stone RJ, et al.,. Effectiveness of technology-enhanced simulation in teaching digital rectal examination: a systematic review narrative synthesis. BMJ Simul Technol Enhanc Learn 2020 Aug 1; 7(5):414–421. PMID: 35515732.

72. Berg MN, Ngune I, Schofield P, et al.,. Effectiveness of online communication skills training for cancer and palliative care health professionals: a systematic review. Psychooncology 2021 Sep; 30(9):1405–1419. PMID: 33909328.

73. Chae D, Yoo JY, Kim J, et al.,. Effectiveness of virtual simulation to enhance cultural competence in pre-licensure and licensed health professionals: a systematic review. Clin Simul Nurs 2021; 56:137–154.

74. Woon APN, Mok WQ, Chieng YJS, et al.,. Effectiveness of virtual reality training in improving knowledge among nursing students: a systematic review, meta-analysis and meta-regression. Nurse Educ Today 2021; 98:104655. PMID: 33303246.

75. Yogeswaran V, El Morr C,. Effectiveness of online mindfulness interventions on medical students' mental health: a systematic review. BMC Public Health 2021 Dec 18; 21(1):2293–z. PMID: 34920715.

76. Berry MCdC, de M Neto JM, de Souza MIdC, et al.,. Effectiveness of technology-enhanced learning to improve periodontics educational outcomes: a systematic review. J Dent Educ 2020 Jul; 84(7):830–839. PMID: 32421234.

77. Khalaf K, El-Kishawi M, Mustafa S, et al.,. Effectiveness of technology-enhanced teaching and assessment methods of undergraduate preclinical dental skills: a systematic review of randomized controlled clinical trials. BMC Med Educ 2020; 20(1):286. PMID: 32859187.

78. Lapierre A, Bouferguene S, Gauvin-Lepage J, et al.,. Effectiveness of interprofessional manikin-based simulation training on teamwork among real teams during trauma resuscitation in adult emergency departments: a systematic review. Simul Healthc 2020; 15(6):409–421. PMID: 32218090.

79. O'Brien B, Bevan K, Brockington C, et al.,. Effects of simulation-based cardiopulmonary and respiratory case training experiences on interprofessional teamwork: a systematic review. Can J Respir Ther 2023 Mar 28; 59:85–94. PMID: 36999003.

80. Lei Y, Zhu L, Sa YTR, et al.,. Effects of high-fidelity simulation teaching on nursing students' knowledge, professional skills and clinical ability: a meta-analysis and systematic review. Nurse Educ Pract 2022; 60:103306. PMID: 35202957.

81. Susilawati E, Johari A, Marzal J, et al.,. Effects of multimedia e-books and augmented reality on knowledge and skills of health sciences students: a systematic review. J Client Centered Nurs Care&nbsp; 2022; 8(2):99–110.

82. Chen B, Wang Y, Xiao L, et al.,. Effects of mobile learning for nursing students in clinical education: a meta-analysis. Nurse Educ Today 2021; 97:104706. PMID: 33360012.

83. Polce EM, Kunze KN, Williams BT, et al.,. Efficacy and validity of orthopaedic simulators in surgical training: a systematic review and meta-analysis of randomized controlled trials. J Am Acad Orthop Surg 2020 Dec 15; 28(24):1027–1040. PMID: 32398408.

84. Woodhead NJ, Mahmud A, Clark J,. Effects of simulation for gynaecological ultrasound scan training: a systematic review. BMJ Simul Technol Enhanc Learn 2020 Nov 1; 6(6):320–331. PMID: 35515489.

85. Maheu-Cadotte M, Cossette S, Dubé V, et al.,. Efficacy of serious games in healthcare professions education: a systematic review and meta-analysis. Simul Healthc 2021; 16(3):199–212. PMID: 33196609.

86. Muirhead K, Macaden L, Smyth K, et al.,. Establishing the effectiveness of technology-enabled dementia education for health and social care practitioners: a systematic review. Syst Rev 2021; 10(1):252. PMID: 34548101.

87. Banks L, Kay R,. Exploring flipped classrooms in undergraduate nursing and health science: a systematic review. Nurse Educ Pract 2022 Oct; Oct(64):103417. PMID: 36113354.

88. Chawla S, Devi S, Calvachi P, et al.,. Evaluation of simulation models in neurosurgical training according to face, content, and construct validity: a systematic review. Acta Neurochir (Wien) 2022 Apr; 164(4):947–966. PMID: 35122126.

89. Chen IA, Ghazi A, Sridhar A, et al.,. Evolving robotic surgery training and improving patient safety, with the integration of novel technologies. World J Urol 2021 Aug; 39(8):2883–2893. PMID: 33156361.

90. Olexa J, Cohen J, Alexander T, et al.,. Expanding educational frontiers in neurosurgery: current and future uses of augmented reality. Neurosurgery 2023; 92(2):241–250. PMID: 36637263.

91. Iop A, El-Hajj VG, Gharios M, et al.,. Extended reality in neurosurgical education: a systematic review. Sensors (Basel) 2022 Aug 14; 22(16):6067. doi: 10.3390/s22166067. PMID: 36015828.

92. Esteban RJR, Juan S. López-McCormick, Alejandra S. Rodríguez-Bermeo, et al.,. Face, content, and construct validity evaluation of simulation models in general surgery laparoscopic training and education: a systematic review. Surg Innov 2023; 30(2):251–260. PMID: 36062557.

93. Youhasan P, Chen Y, Lyndon M, et al.,. Exploring the pedagogical design features of the flipped classroom in undergraduate nursing education: a systematic review. BMC Nurs 2021; 20(1):50. PMID: 33752654.

94. Li B, Cao N, Ren C, et al.,. Flipped classroom improves nursing students' theoretical learning in China: a meta-analysis. PLoS One 2020 Aug 27; 15(8):e0237926. PMID: 32853214.

95. van Gaalen AEJ, Brouwer J, Schönrock-Adema J, et al.,. Gamification of health professions education: a systematic review. Adv Health Sci Educ Theory Pract 2021 May; 26(2):683–711. PMID: 33128662.

96. Berthold DP, Muench LN, Rupp M, et al.,. Head-mounted display virtual reality is effective in orthopaedic training: a systematic review. Arthrosc Sports Med Rehabil 2022 Aug 2; 4(5):e1843–e1849. PMID: 36312724.

97. Corvetto MA, Altermatt FR, Belmar F, et al.,. Health care simulation as a training tool for epidemic management: a systematic review. Simul Healthc 2023; 18(6):382–391. PMID: 36881436.

98. Li YY, Au ML, Tong LK, et al.,. High-fidelity simulation in undergraduate nursing education: A meta-analysis. Nurse Educ Today 2022; Apr(111):105291. PMID: 35158134.

99. Orejuela FJ, Aschkenazi SO, Howard DL, et al.,. Gynecologic surgical skill acquisition through simulation with outcomes at the time of surgery: a systematic review and meta-analysis. Am J Obstet Gynecol 2022; 227(1):29.e1–29.e24. PMID: 35120886.

100. Astbury J, Ferguson J, Silverthorne J, et al.,. High-fidelity simulation-based education in pre-registration healthcare programmes: a systematic review of reviews to inform collaborative and interprofessional best practice. J Interprof Care 2021; 35(4):622–632. PMID: 32530344.

101. Lowe S, Mares K, Khadjesari Z,. Immersive technology in ophthalmology education: a systematic review. BMJ Simul Technol Enhanc Learn 2021 Jun 29; 7(6):600–604. PMID: 35520978.

102. Mao RQ, Lan L, Kay J, et al.,. Immersive virtual reality for surgical training: a systematic review. J Surg Res 2021; Dec(268):40–58. PMID: 34284320.

103. Le Lous M, Simon O, Lassel L, et al.,. Hybrid simulation for obstetrics training: a systematic review. Eur J Obstet Gynecol Reprod Biol 2020 Mar; Mar(246):23–28. PMID: 31927239.

104. Au ML, Tong LK, Li YY, et al.,. Impact of scenario validity and group size on learning outcomes in high-fidelity simulation: a systematics review and meta-analysis. Nurse Educ Today 2023; Feb(121):105705. PMID: 36599250.

105. Adewuyi M, Morales K, Lindsey A,. Impact of experiential dementia care learning on knowledge, skills and attitudes of nursing students: a systematic literature review. Nurse Educ Pract 2022 Jul; Jul(62):103351. PMID: 35483217.

106. Chan SCC, Choa G, Kelly J, et al.,. Implementation of virtual OSCE in health professions education: a systematic review. Med Educ 2023 -09; 57(9):833–843. PMID: 37080907.

107. Oliveira Silva G, Oliveira FSE, Coelho ASG, et al.,. Influence of simulation design on stress, anxiety and self-confidence of nursing students: systematic review with meta-analysis. J Clin Nurs 2023 -09; 32(17-18):5668–5692. PMID: 36894868.

108. Gawronski O, Thekkan KR, Genna C, et al.,. Instruments to evaluate non-technical skills during high fidelity simulation: a systematic review. Front Med (Lausanne) 2022 November 3; 9:986296. PMID: 36405618.

109. Wirth T, Peters C, Nienhaus A, et al.,. Interventions for workplace violence prevention in emergency departments: a systematic review. Int J Environ Res Public Health 2021 Aug 10; 18(16):8459. doi: 10.3390/ijerph18168459. PMID: 34444208.

110. Mao BP, Teichroeb ML, Lee T, et al.,. Is online video-based education an effective method to teach basic surgical skills to students and surgical trainees? A systematic review and meta-analysis. J Surg Educ 2022; 79(6):1536–1545. PMID: 35933308.

111. Ryan GV, Callaghan S, Rafferty A, et al.,. Learning outcomes of immersive technologies in health care student education: systematic review of the literature. J Med Internet Res 2022 Feb 1; 24(2):e30082. PMID: 35103607.

112. Sleiman Z, Bitar R, Christoforou C, et al.,. Is there proof of transferability of laparoscopic psychomotor skills from the simulator laboratory to the operating room? Results from a systematic review. J Obstet Gynaecol 2022 Feb; 42(2):181–187. PMID: 34027781.

113. Xu Y, Lau Y, Cheng LJ, et al.,. Learning experiences of game-based educational intervention in nursing students: a systematic mixed-studies review. Nurse Educ Today 2021; Dec(107):105139. PMID: 34563963.

114. Lam K, Chen J, Wang Z, et al.,. Machine learning for technical skill assessment in surgery: a systematic review. NPJ Digit Med 2022; 5(1):24.

115. Tabatabaeichehr M, Babaei S, Dartomi M, et al.,. Medical students’ satisfaction level with e-learning during the COVID-19 pandemic and its related factors: a systematic review. J Educ Eval Health Prof 2022; 19:37. PMID: 36579447.

116. Dedeilia A, Sotiropoulos MG, Hanrahan JG, et al.,. Medical and surgical education challenges and innovations in the COVID-19 era: a systematic review. In Vivo 2020 Jun; 34(3 Suppl):1603–1611. PMID: 32503818.

117. Chandran VP, Balakrishnan A, Rashid M, et al.,. Mobile applications in medical education: a systematic review and meta-analysis. PLoS One 2022 Mar 24; 17(3):e0265927. PMID: 35324994.

118. Margulies IG, Xu H, Henderson PW,. Microsurgery training in the digital era: a systematic review of accessible digital resources. Ann Plast Surg 2020 Oct; 85(4):337–343. PMID: 31923014.

119. Chen J, Xun H, Abousy M, et al.,. No microscope? No problem: a systematic review of microscope-free microsurgery training models. J Reconstr Microsurg 2021; 38(02):106–114. PMID: 34425592.

120. Shorey S, Pereira TL, TEO WZ, et al.,. Navigating nursing curriculum change during COVID-19 pandemic: a systematic review and meta-synthesis. Nurse Educ Pract 2022; Nov(65):103483. PMID: 36327596.

121. Stojan J, Haas M, Thammasitboon S, et al.,. Online learning developments in undergraduate medical education in response to the COVID-19 pandemic: a BEME systematic review: BEME Guide No. 69. Med Teach 2022; 44(2):109–129. PMID: 34709949.

122. Castillo-Segura P, Fernández-Panadero C, Alario-Hoyos C, et al.,. Objective and automated assessment of surgical technical skills with IoT systems: a systematic literature review. Artif Intell Med 2021; Feb(112):102007. PMID: 33581827.

123. Anugrahsari S, Chaeruman UA, Abbas H, et al.,. Patient safety education for clinical students: a systematic literature review. Open Access Maced J Med Sci 2022; 10(F):208–214.

124. Chan RJ, Agbejule OA, Yates PM, et al.,. Outcomes of cancer survivorship education and training for primary care providers: a systematic review. J Cancer Surviv 2022; 16(2):279–302. PMID: 33763806.

125. Schafer KM, Kremer MJ,. Outcomes of simulation-based experiences related to family presence during resuscitation: a systematic review. Clin Simul Nurs 2022; 65:62–81.

126. Tudor Car L, Kyaw BM, Teo A, et al.,. Outcomes, measurement instruments, and their validity evidence in randomized controlled trials on virtual, augmented, and mixed reality in undergraduate medical education: systematic mapping review. JMIR Serious Games 2022; 10(2):e29594. PMID: 35416789.

127. Okano H, Mayumi T, Kataoka Y, et al.,. Outcomes of simulation-based education for vascular access: a systematic review and meta-analysis. Cureus 2021; 13(8):e17188. PMID: 34414052.

128. Grafton-Clarke C, Uraiby H, Gordon M, et al.,. Pivot to online learning for adapting or continuing workplace-based clinical learning in medical education following the COVID-19 pandemic: A BEME systematic review: BEME Guide No. 70. Med Teach 2022; 44(3):227–243. PMID: 34689692.

129. Oh S, Cook DA, Van Gerven PWM, et al.,. Physician training for electrocardiogram interpretation: a systematic review and meta-analysis. Acad Med 2022; 97(4):593–602. PMID: 35086115.

130. Pires C,. Perceptions of pharmacy students on the e-learning strategies adopted during the covid-19 pandemic: a systematic review. Pharmacy (Basel) 2022 Feb 15; 10(1):31. doi: 10.3390/pharmacy10010031. PMID: 35202080.

131. Baetzner AS, Wespi R, Hill Y, et al.,. Preparing medical first responders for crises: a systematic literature review of disaster training programs and their effectiveness. Scand J Trauma Resusc Emerg Med 2022; 30(1):76. PMID: 36566227.

132. Savage AJ, McNamara PW, Moncrieff TW, et al.,. Review article: e-learning in emergency medicine: a systematic review. Emerg Med Australas 2022; 34(3):322–332. PMID: 35224870.

133. Legoux C, Gerein R, Boutis K, et al.,. Retention of critical procedural skills after simulation training: a systematic review. AEM Educ Train 2021; 5(3):e10536. PMID: 34099989.

134. A. A. Tori, R. Tori, F. d. L. d. S. Nunes,. Serious game design in health education: a systematic review. IEEE T Learn Technol 2022; 15(6):827–846.

135. Nayar SK, Musto L, Baruah G, et al.,. Self-assessment of surgical skills: a systematic review. &nbsp;J Surg Educ 2020; 77(2):348–361. PMID: 31582350.

136. Bray L, Krogh TB, Østergaard D,. Simulation-based training for continuing professional development within a primary care context: a systematic review. Educ Prim Care 2023; 34(2):64–73. PMID: 36730551.

137. Richard O, Jollant F, Billon G, et al.,. Simulation training in suicide risk assessment and intervention: a systematic review and meta-analysis. Med Educ Online 2023 Dec; 28(1):2199469. PMID: 37073473.

138. Chou WK, Ullah N, Arjomandi Rad A, et al.,. Simulation training for obstetric emergencies in low- and lower-middle income countries: a systematic review. &nbsp;Eur J Obstet Gynecol Reprod Biol 2022; Sep(276):74–81. PMID: 35820293.

139. Heuer A, Bienstock J, Zhang Y,. Simulation-based training within selected allied health professions: an evidence-based systematic review. J Allied Health 2022; 51(1):59–71. PMID: 35239763.

140. Tokas T, Ortner G, Peteinaris A, et al.,. Simulation training in transurethral resection/laser vaporization of the prostate; evidence from a systematic review by the European Section of Uro-Technology. World J Urol 2022; 40(5):1091–1110. PMID: 34800135.

141. Zaed I, Chibbaro S, Ganau M, et al.,. Simulation and virtual reality in intracranial aneurysms neurosurgical training: a systematic review. J Neurosurg Sci 2022 Dec; 66(6):494–500. PMID: 35301837.

142. Aydın A, Baig U, Al-Jabir A, et al.,. Simulation-based training models for urolithiasis: a systematic review. J Endourol 2021; 35(7):1098–1117. PMID: 33198492.

143. Davids J, Manivannan S, Darzi A, et al.,. Simulation for skills training in neurosurgery: a systematic review, meta-analysis, and analysis of progressive scholarly acceptance. Neurosurg Rev 2021 Aug; 44(4):1853–1867. PMID: 32944808.

144. Lindhard MS, Thim S, Laursen HS, et al.,. Simulation-based neonatal resuscitation team training: a systematic review. Pediatrics 2021; 147(4):e2020042010. PMID: 33762309.

145. Tropea J, Bicknell R, Nestel D, et al.,. Simulation training in non-cancer palliative care for healthcare workers: a systematic review of controlled studies. BMJ Simul Technol Enhanc Learn 2020 Aug 13; 7(4):262–269. PMID: 35516824.

146. Nielsen CA, Lönn L, Konge L, et al.,. Simulation-based virtual-reality patient-specific rehearsal prior to endovascular procedures: a systematic review. Diagnostics (Basel) 2020 Jul 20; 10(7):500. doi: 10.3390/diagnostics10070500. PMID: 32698437.

147. Sultana N, Betran AP, Khan KS, et al.,. Simulation-based teaching and models for caesarean sections: a systematic review to evaluate the tools for the ‘See One, Practice Many, Do One’ slogan. Curr Opin Obstet Gynecol 2020; 32(5):305–315. PMID: 32796165.

148. Fouasson-Chailloux A, Daley P, Menu P, et al.,. Social media in health studies: a systematic review of comparative learning methods. Int J Environ Res Public Health 2022 Feb 15; 19(4):2205. doi: 10.3390/ijerph19042205. PMID: 35206401.

149. Pietersen PI, Bjerrum F, Tolsgaard MG, et al.,. Standard setting in simulation-based training of surgical procedures: a systematic review. Ann Surg 2022; 275(5):872–882. PMID: 34520423.

150. Guckian J, Utukuri M, Asif A, et al.,. Social media in undergraduate medical education: a systematic review. Med Educ 2021 Nov; 55(11):1227–1241. PMID: 33988867.

151. Jacob C, Sanchez-Vazquez A, Ivory C,. Social, organizational, and technological factors impacting clinicians’ adoption of mobile health tools: systematic literature review. JMIR Mhealth Uhealth 2020; 8(2):e15935. PMID: 32130167.

152. Jones A, Wilson I, McClean S, et al.,. Supporting the learning experience of health-related profession students during clinical placements with technology: a systematic review. Rev Educ 2022; 10(2):e3364.

153. Rangarajan K, Davis H, Pucher PH,. Systematic review of virtual haptics in surgical simulation: a valid educational tool?. J Surg Educ 2020; 77(2):337–347. PMID: 31564519.

154. Rashidian N, Vierstraete M, Alseidi A, et al.,. Surgical education interventions in liver surgery: a systematic review. Updates Surg 2020; 72(3):583–594. PMID: 32342345.

155. Hildreth AF, Maggio LA, Iteen A, et al.,. Technology-enhanced simulation in emergency medicine: updated systematic review and meta-analysis 1991–2021. AEM Educ Train 2023; 7(2):e10848. PMID: 36936085.

156. Santos VA, Barreira MP, Saad KR,. Technological resources for teaching and learning about human anatomy in the medical course: systematic review of literature. Anat Sci Educ 2022; 15(2):403–419. PMID: 34664384.

157. Jin C, Dai L, Wang T,. The application of virtual reality in the training of laparoscopic surgery: a systematic review and meta-analysis. Int Surg J 2021; Mar(87):105859. PMID: 33307244.

158. Neubauer R, Recker F, Bauer CJ, et al.,. The current situation of musculoskeletal ultrasound education: a systematic literature review. Ultrasound Med Biol 2023; 49(6):1363–1374. PMID: 36941182.

159. Muirhead K, Macaden L, Smyth K, et al.,. The characteristics of effective technology-enabled dementia education: a systematic review and mixed research synthesis. Syst Rev 2022; 11(1):34. PMID: 35197109.

160. Ozdemir NG, Kaya H,. The effect of high-fidelity simulation on experiences of urinary catheterization in nursing students: a mixed-method systematic review. Int J Caring Sci 2022 May; 15(2):1218–1232.

161. Brown L, Ilhan E, Pacey V, et al.,. The effect of high-fidelity simulation–based learning in acute cardiorespiratory physical therapy: a mixed-methods systematic review. J Phys Ther 2021; 35(2):146–158.

162. Helming AG, Adler DS, Keltner C, et al.,. The content quality of YouTube videos for professional medical education: a systematic review. Acad Med 2021; 96(10):1484–1493. PMID: 33856363.

163. Fadzilah Hanum MM, Choo WY, Othman S,. The effectiveness of educational intervention in improving primary health-care service providers’ knowledge, identification, and management of elder abuse and neglect: a systematic review. Trauma Violence Abuse 2021; 22(4):944–960. PMID: 31823685.

164. Sinacori B, Williams-Gregory M,. The effect of distance learning on knowledge acquisition in undergraduate second-degree nursing students: a systematic review. Nurs Educ Perspect 2021; 42(3):136–141. PMID: 33323720.

165. Svellingen AH, Søvik MB, Røykenes K, et al.,. The effect of multiple exposures in scenario-based simulation: a mixed study systematic review. Nurs Open 2021; 8(1):380–394. PMID: 33318846.

166. Taba JV, Cortez VS, Moraes WA, et al.,. The development of laparoscopic skills using virtual reality simulations: a systematic review. PLoS One 2021 Jun 17; 16(6):e0252609. PMID: 34138901.

167. Zhao G, Fan M, Yuan Y, et al.,. The comparison of teaching efficiency between virtual reality and traditional education in medical education: a systematic review and meta-analysis. Ann Transl Med 2021 Feb; 9(3):252–2785. PMID: 33708879.

168. Alshehri FD, Jones S, Harrison D,. The effectiveness of high-fidelity simulation on undergraduate nursing students' clinical reasoning-related skills: a systematic review. Nurse Educ Today 2023; Feb(121):105679. PMID: 36542870.

169. Zhao J, Xu X, Jiang H, et al.,. The effectiveness of virtual reality-based technology on anatomy teaching: a meta-analysis of randomized controlled studies. BMC Med Educ 2020; 20(1):127. PMID: 32334594.

170. Jallad ST, Işık B,. The effectiveness of virtual reality simulation as learning strategy in the acquisition of medical skills in nursing education: a systematic review. Ir J Med Sci 2022; 191(3):1407–1426. PMID: 34227032.

171. Plackett R, Kassianos AP, Mylan S, et al.,. The effectiveness of using virtual patient educational tools to improve medical students’ clinical reasoning skills: a systematic review. BMC Med Educ 2022; 22(1):365. PMID: 35550085.

172. Yucel C, Hawley G, Terzioglu F, et al.,. The effectiveness of simulation-based team training in obstetrics emergencies for improving technical skills: a systematic review. Simul Healthc 2020; 15(2):98–105. PMID: 32168287.

173. Capitani P, Zampogna B, Monaco E, et al.,. The role of virtual reality in knee arthroscopic simulation: a systematic review. Musculoskelet Surg 2023; 107(1):85–95. PMID: 34655024.

174. Gomaa A, Grafton-Clarke C, Saratzis A, et al.,. The role of high-fidelity simulation in the acquisition of endovascular surgical skills: a systematic review. Ann Vasc Surg 2023 Jul; Jul(93):405–427. PMID: 36906131.

175. Howlader D, Daga D, Mehrotra D,. The scope of computerized simulation in competency-based maxillofacial training: a systematic review. Int J Oral Maxillofac Surg 2022; 51(8):1101–1110. PMID: 35058106.

176. Suresh D, Aydin A, James S, et al.,. The role of augmented reality in surgical training: a systematic review. Surg Innov 2023; 30(3):366–382. PMID: 36412148.

177. Santos GNM, da Silva HEC, Leite AF, et al.,. The scope of dental education during COVID-19 pandemic: a systematic review. J Dent Educ 2021; 85(7):1287–1300. PMID: 33759214.

178. van Schaijik B, Alshawa A, Hamadah O, et al.,. The role of Twitter in dental education: a systematic review. J Dent Educ 2021; 85(9):1471–1481. PMID: 33948963.

179. Srinivasa K, Chen Y, Henning MA,. The role of online videos in teaching procedural skills to post-graduate medical learners: a systematic narrative review. Med Teach 2020; 42(6):689–697. PMID: 32174211.

180. Almutairi M, Simpson A, Khan E, et al.,. The value of social media use in improving nursing students’ engagement: a systematic review. Nurse Educ Pract 2022; Oct(64):103455. PMID: 36182729.

181. Shorey S, Ng ED,. The use of virtual reality simulation among nursing students and registered nurses: a systematic review. Nurse Educ Today 2021; Mar(98):104662. PMID: 33203545.

182. Chahal B, Aydın A, Amin MSA, et al.,. Transfer of open and laparoscopic skills to robotic surgery: a systematic review. J Robot Surg 2023; 17(4):1207–1225. PMID: 36418717.

183. Fahl JT, Duvivier R, Reinke L, et al.,. Towards best practice in developing motor skills: a systematic review on spacing in VR simulator-based psychomotor training for surgical novices. BMC Med Educ 2023; 23(1):154. PMID: 36907871.

184. O'Connor S, Kennedy S, Wang Y, et al.,. Theories informing technology enhanced learning in nursing and midwifery education: a systematic review and typological classification. Nurse Educ Today 2022; Nov(118):105518. PMID: 36030581.

185. Jujo S, Nakahira A, Kataoka Y, et al.,. Transesophageal echocardiography simulator training: a systematic review and meta-analysis of randomized controlled trials. Simul Healthc 2021; 16(5):341–352. PMID: 33428355.

186. Smith SCL, Siau K, Cannatelli R, et al.,. Training methods in optical diagnosis and characterization of colorectal polyps: a systematic review and meta-analysis. Endosc Int Open 2021 May; 9(5):E716–E726. PMID: 33937513.

187. Engberg M, Taudorf M, Rasmussen NK, et al.,. Training and assessment of competence in resuscitative endovascular balloon occlusion of the aorta (REBOA) — a systematic review. Injury 2020; 51(2):147–156. PMID: 31810637.

188. Cant R, Ryan C, Kelly MA,. Use and effectiveness of virtual simulations in nursing student education: an umbrella review. Comput Inform Nurs 2023; 41(1):31–38. PMID: 35926237.

189. Stone R, Cooke M, Mitchell M,. Undergraduate nursing students' use of video technology in developing confidence in clinical skills for practice: a systematic integrative literature review. Nurse Educ Today 2020; 84:104230. PMID: 31689584.

190. Ayivi-Vinz G, Bakwa Kanyinga F, Bergeron L, et al.,. Use of the CPD-REACTION questionnaire to evaluate continuing professional development activities for health professionals: systematic review. JMIR Med Educ 2022 May 2; 8(2):e36948. PMID: 35318188.

191. ShahAli S, Shahabi S, Kohan N, et al.,. Using e-learning methods for physiotherapy students learning – a systematic review and meta-analysis of the impact on knowledge, skills, satisfaction and attitudes. Eur J Physiother 2023; 25(5):252–264.

192. Verville L, Dc PC, Grondin D, et al.,. Using technology-based educational interventions to improve knowledge about clinical practice guidelines. J Chiropr Educ 2021 Mar 1; 35(1):149–157. PMID: 32931558.

193. Garnier A, Vanherp R, Bonnabry P, et al.,. Use of simulation for education in hospital pharmaceutical technologies: a systematic review. Eur J Hosp Pharm 2023; 30(2):70–76. PMID: 34949651.

194. Singh T, Reyes-Portillo JA,. Using technology to train clinicians in evidence-based treatment: a systematic review. Psychiatr Serv 2020 Apr 1; 71(4):364–377. PMID: 31960775.

195. Daniel R, McKechnie T, Kruse CC, et al.,. Video-based coaching for surgical residents: a systematic review and meta-analysis. Surg Endosc 2023; 37(2):1429–1439. PMID: 35739431.

196. Tursø-Finnich T, Jensen RO, Jensen LX, et al.,. Virtual reality head-mounted displays in medical education: a systematic review. Simul Healthc 2023; 18(1):42–50. PMID: 35136005.

197. Beshir SA, Mohamed AP, Soorya A, et al.,. Virtual patient simulation in pharmacy education: a systematic review. Pharm Educ 2022; 22(1):p. 954–970.

198. Dzyuba N, Jandu J, Yates J, et al.,. Virtual and augmented reality in dental education: the good, the bad and the better. EurJ Dent Educ 2022; n/a PMID: 36336847.

199. Lucena-Anton D, Fernandez-Lopez J, Pacheco-Serrano A, et al.,. Virtual and augmented reality versus traditional methods for teaching physiotherapy: a systematic review. Eur J Investig Health Psychol Educ 2022; 12(12):1792. PMID: 36547026.

200. Umphrey L, Lenhard N, Lam SK, et al.,. Virtual global health in graduate medical education: a systematic review. Int J Med Educ 2022; 13:230–248. PMID: 36057978.

201. Gelmini AYP, Duarte ML, de Assis AM, et al.,. Virtual reality in interventional radiology education: a systematic review. Radiol Bras 2021; 54(4):254–260. PMID: 34393293.

202. Kovoor JG, Gupta AK, Gladman MA,. Validity and effectiveness of augmented reality in surgical education: a systematic review. Surgery 2021; 170(1):88–98. PMID: 33744003.

203. Moro C, Birt J, Stromberga Z, et al.,. Virtual and augmented reality enhancements to medical and science student physiology and anatomy test performance: a systematic review and meta-analysis. Anat Sci Educ 2021; 14(3):368–376. PMID: 33378557.

204. Lohre R, Wang JC, Lewandrowski K, et al.,. Virtual reality in spinal endoscopy: a paradigm shift in education to support spine surgeons. J Spine Surg 2020 Jan; 6(Suppl 1):S208–S223. PMID: 32195429.

205. Richardson CL, White S, Chapman S,. Virtual patient technology to educate pharmacists and pharmacy students on patient communication: a systematic review. BMJ Simul Technol Enhanc Learn 2020 Nov 1; 6(6):332–338. PMID: 35515492.

206. Asadzandi S, Mojtahedzadeh R, Mohammadi A,. What are the factors that enhance metacognitive skills in nursing students? A systematic review. Iran J Nurs Midwifery Res 2022 Nov 18; 27(6):475–484. PMID: 36712305.

207. Brown N, Margus C, Hart A, et al.,. Virtual reality training in disaster medicine: a systematic review of the literature. Simul Healthc 2023; 18(4):255–261. PMID: 35696131.

208. Clerkin R, Patton D, Moore Z, et al.,. What is the impact of video as a teaching method on achieving psychomotor skills in nursing? A systematic review and meta-analysis. Nurse Educ Today 2022; Apr(111):105280. PMID: 35139443.

209. Sim JJM, Rusli KDB, Seah B, et al.,. Virtual simulation to enhance clinical reasoning in nursing: a systematic review and meta-analysis. Clin Simul Nurs 2022 Aug; Aug(69):26–39. PMID: 35754937.

210. Clarke E,. Virtual reality simulation—the future of orthopaedic training? A systematic review and narrative analysis. Adv Simul (Lond) 2021; 6(1):2. PMID: 33441190.

211. Lin JC, Yu Z, Scott IU, et al.,. Virtual reality training for cataract surgery operating performance in ophthalmology trainees. Cochrane Database Syst Rev 2021; 12(12):CD014953. PMID: 34931701.

212. Plotzky C, Lindwedel U, Sorber M, et al.,. Virtual reality simulations in nurse education: a systematic mapping review. Nurse Educ Today 2021; Jun(101):104868. PMID: 33798987.

213. Schmidt MW, Köppinger KF, Fan C, et al.,. Virtual reality simulation in robot-assisted surgery: meta-analysis of skill transfer and predictability of skill. BJS Open 2021; 5(2):zraa066. PMID: 33864069.

214. Tolarba JEL,. Virtual simulation in nursing education: a systematic review. In J Nurs Educ 2021 Jul; 13(3):48–54. PMID: 32028447.

215. Foronda CL, Fernandez-Burgos M, Nadeau C, et al.,. Virtual simulation in nursing education: a systematic review spanning 1996 to 2018. Simul Healthc 2020; 15(1):46–54. PMID: 32028447.

216. Portelli M, Bianco SF, Bezzina T, et al.,. Virtual reality training compared with apprenticeship training in laparoscopic surgery: a meta-analysis. Ann R Coll Surg Engl 2020; 102(9):672–684. PMID: 32820649.

217. Lee W, Kim G, Yoon HJ, et al.,. A systematic review of the effect of web-based trauma-education programs for mental-health professionals. J Korean Acad Psychiatr Ment Health Nurs 2020; 29(4):325–338.
